# Therapy-induced lipid uptake and remodeling underpin ferroptosis hypersensitivity in prostate cancer

**DOI:** 10.1186/s40170-020-00217-6

**Published:** 2020-06-19

**Authors:** Kaylyn D. Tousignant, Anja Rockstroh, Berwyck L. J. Poad, Ali Talebi, Reuben S. E. Young, Atefeh Taherian Fard, Rajesh Gupta, Tuo Zang, Chenwei Wang, Melanie L. Lehman, Johan V. Swinnen, Stephen J. Blanksby, Colleen C. Nelson, Martin C. Sadowski

**Affiliations:** 1 Australian Prostate Cancer Research Centre - Queensland, Institute of Health and Biomedical Innovation, School of Biomedical Sciences, Faculty of Health, Queensland University of Technology (QUT), Princess Alexandra Hospital, Translational Research Institute, Brisbane, Australia; 2 Cancer & Ageing Research Program, Institute of Health and Biomedical Innovation, School of Biomedical Sciences, Faculty of Health, Queensland University of Technology (QUT), Translational Research Institute, Brisbane, Australia; 3 Central Analytical Research Facility, Institute for Future Environments, Queensland University of Technology, Brisbane, Australia; 4 Department of Oncology, Laboratory of Lipid Metabolism and Cancer, LKI Leuven Cancer Institute, KU Leuven-University of Leuven, Leuven, Belgium; 5 Vancouver Prostate Centre, Department of Urologic Sciences, University of British Columbia, Vancouver, Canada

**Keywords:** Therapy resistance, Multidrug tolerance, Metabolic reprograming, Lipid remodeling, Lipid uptake, Ferroptosis, GPX4, Prostate cancer

## Abstract

**Background:**

Metabolic reprograming, non-mutational epigenetic changes, increased cell plasticity, and multidrug tolerance are early hallmarks of therapy resistance in cancer. In this temporary, therapy-tolerant state, cancer cells are highly sensitive to ferroptosis, a form of regulated cell death that is caused by oxidative stress through excess levels of iron-dependent peroxidation of polyunsaturated fatty acids (PUFA). However, mechanisms underpinning therapy-induced ferroptosis hypersensitivity remain to be elucidated.

**Methods:**

We used quantitative single-cell imaging of fluorescent metabolic probes, transcriptomics, proteomics, and lipidomics to perform a longitudinal analysis of the adaptive response to androgen receptor-targeted therapies (androgen deprivation and enzalutamide) in prostate cancer (PCa).

**Results:**

We discovered that cessation of cell proliferation and a robust reduction in bioenergetic processes were associated with multidrug tolerance and a strong accumulation of lipids. The gain in lipid biomass was fueled by enhanced lipid uptake through cargo non-selective (macropinocytosis, tunneling nanotubes) and cargo-selective mechanisms (lipid transporters), whereas de novo lipid synthesis was strongly reduced. Enzalutamide induced extensive lipid remodeling of all major phospholipid classes at the expense of storage lipids, leading to increased desaturation and acyl chain length of membrane lipids. The rise in membrane PUFA levels enhanced membrane fluidity and lipid peroxidation, causing hypersensitivity to glutathione peroxidase (GPX4) inhibition and ferroptosis. Combination treatments against AR and fatty acid desaturation, lipase activities, or growth medium supplementation with antioxidants or PUFAs altered GPX4 dependence.

**Conclusions:**

Our work provides mechanistic insight into processes of lipid metabolism that underpin the acquisition of therapy-induced GPX4 dependence and ferroptosis hypersensitivity to standard of care therapies in PCa. It demonstrates novel strategies to suppress the therapy-tolerant state that may have potential to delay and combat resistance to androgen receptor-targeted therapies, a currently unmet clinical challenge of advanced PCa. Since enhanced GPX4 dependence is an adaptive phenotype shared by several types of cancer in response to different therapies, our work might have universal implications for our understanding of metabolic events that underpin resistance to cancer therapies.

## Background

Despite significant advancements in detection and treatment over the past decades, prostate cancer (PCa) remains the second most commonly diagnosed cancer among men and the third leading cause of cancer mortality in men worldwide [1]. A major challenge in PCa management stems not from the lack of initial treatment options, but in therapy adaptation leading to resistance. Given the dependence of PCa cells on androgens for growth, proliferation, survival, and differentiation, the primary treatment for men with advanced PCa is androgen deprivation therapy (ADT), which is being increasingly supplemented with a new generation of potent androgen synthesis inhibitors (abiraterone) or androgen receptor (AR) antagonists bicalutamide, enzalutamide, and apalutamide [2, 3], hereafter cumulatively termed androgen-targeted therapies (ATTs). Despite initial treatment response, unfortunately ATTs will ultimately fail in most PCa patients, and disease progresses to castrate-resistant PCa (CRPC) [3, 4] in which PCa cells have often adopted a number of complex resistance mechanisms, including AR amplification, mutation, or splice variants to maintain AR function in the low androgen environment [3, 5]. Other resistance mechanisms bypass AR by exploiting alternative signaling and metabolic pathways. Understanding the early underlying molecular mechanisms that ultimately result in resistance to ATTs is critical for the discovery and development of co-treatment strategies and the definition of therapeutic windows to extend the efficacy of ATTs and combat therapy resistance.

While previous studies in PCa provided an extensive characterization of PCa models with established ATT resistance [6–8], therapy-induced early metabolic reprogramming that underpins therapy survival and development of drug resistance has remained largely unexplored. Recent work in breast, melanoma, lung, and ovarian cancer showed that different targeted anti-cancer therapies induced a transient quiescent state of negligible growth termed drug-induced persister cells [9]. Persister cells are characterized by increased expression of markers of dedifferentiation, enhanced lipid peroxidation, an acquired dependency on lipid hydroperoxidase GPX4, and sensitivity to ferroptosis that offer novel therapeutic strategies to combat resistance [9]. Whether ATTs cause PCa cells to acquire the persister cell phenotype remains to be shown.

Ferroptosis is a form of regulated cell death characterized by iron-dependent lipid peroxidation of polyunsaturated fatty acids (PUFAs) that was recently identified as a metabolic vulnerability in several types of cancer, including lymphoma, renal cell carcinoma, and breast cancer [10, 11]. In particular, reactive oxygen species-mediated peroxidation of PUFAs arachidonic acid and adrenic acid within the phospholipid class of phosphatidylethanolamines are potent activators of ferroptosis [12]. Glutathione peroxidase 4 (GPX4), a selenocysteine enzyme that utilizes glutathione to reduce toxic lipid peroxides into non-toxic lipid alcohols, is critical for the protection against lipid peroxide-mediated damage and ferroptosis induction [11]. Recently, a second, glutathione-independent protective pathway containing ferroptosis-suppressor-protein- 1 (FSP1/AIFM2) was found to act in parallel to GPX4 [13, 14]. FSP1 functions as an oxidoreductase with coenzyme Q10 at the plasma membrane to generate lipophilic radical-trapping antioxidants that halt lipid peroxidation [13]. While ferroptosis is an attractive and highly promising concept to combat resistance to multiple therapies in various types of cancer, the metabolic changes that lead to enhanced ferroptosis sensitivity in the context of therapy resistance remain uncharacterized [9].

Dysregulation of lipid homeostasis is a metabolic hallmark associated with PCa tumorigenesis and disease progression to CRPC in response to ATTs. Yet, therapy-induced metabolic reprograming and lipid supply mechanisms other than de novo lipid synthesis have not been studied in this context. Previous data from our longitudinal xenograft model of progression to CRPC demonstrated alterations in lipid metabolism during the development to CRPC [15], including increased intratumoral levels of essential PUFAs derived from dietary sources implicating lipid uptake. Emerging evidence reveals lipid scavenging routes utilized by cancer cells during stress in cell culture and pre-clinical models, including lipid transporters [16], the formation of tunneling nanotubes [17], serum albumin-lipid conjugate uptake, and macropinocytosis of necrotic cell debris from the microenvironment [18]. Many of these lipid supply pathways converge in lysosomes, where degradation of endocytic vesicles releases lipids and other nutrients intracellularly [19], highlighting the importance of lysosomal activity for lipid supply.

In the present study, we delineated the longitudinal metabolic changes in PCa cells in response to ATTs that lead to therapy-induced persister cells characterized by increased lipid content and uptake and substantial lipid remodeling, multidrug tolerance and ferroptosis hypersensitivity. We show that acquisition of this therapy-induced phenotype can be efficiently suppressed by several combination treatments directed against lipid processing enzymes. In addition, this work identified enhanced dependence of persister cell on lysosomal processing of exogenous cholesterol. Together, our work gained novel mechanistic insight into therapy-induced ferroptosis hypersensitivity and provided multiple strategies that have the potential to extend the efficacy of ATTs and delay resistance development and disease progression to lethal CRPC.

## Methods

### Cell culture

LNCaP (ATCC, CVCL_0395) and C4-2B (ATCC, CVCL_4784) cells were cultured in RPMI medium (Thermo Fisher) supplemented with 5% FBS. Medium was changed every 3 days and cells were incubated at 37 °C in 5% CO_2_. For treatments representing androgen-deprivation therapy, cells were cultured in RPMI medium supplemented with 5% charcoal -stripped serum (CSS). For AR-targeted therapies, cells were cultured in 5% or 10% FBS with AR antagonist enzalutamide (Enz, 10 μM). Cells were passaged at approximately 80% confluency by trypsinization. Cell lines were genotyped in March 2018 by Genomics Research Centre (Brisbane) and routinely tested for mycoplasma infection.

### RNA extraction and quantitative real-time polymerase chain reaction (qRT-PCR)

Forty-eight hours after seeding, cells were treated with the indicated inhibitors. After an additional 48 h, RNA was harvested using the RNEasy mini kit (Qiagen) following the manufacturer’s instructions. Before extraction, RNA was treated with DNase (Qiagen) to improve RNA purity. Concentration of RNA was measured using a NanoDrop ND-1000 Spectrophotometer (ThermoScientific), and cDNA was prepared from 2 μg total RNA with Superscript III (Invitrogen). qRT-PCR was performed with SYBR Green PCR Master Mix (Invitrogen) on a ViiA-7 Real-Time PCR system (Applied Biosystems). Determination of relative mRNA levels was calculated using the comparative ΔΔCT method [20] compared to the expression of housekeeping gene receptor like protein 32 (*RPL32*) in each treatment and calculated as fold change relative to vehicle control (DMSO). All experiments were performed in triplicate, and analysis and statistics were performed with GraphPad Prism software. Primer sequences can be found in supplementary information (Table S5).

### Microarray gene expression profiling on the 180k VPC custom arrays

For gene expression profiling, triplicates of each sample were analyzed on a custom 180k Agilent oligo microarray (VPCv3, ID032034, GEO: GPL16604). This array contains probes mapping to human protein-coding as well as non-coding loci, with probes targeting exons, 3′UTRs, 5′UTRs, intronic, and intergenic regions. RNA was isolated using the RNeasy Mini Kit (Qiagen, Hilden, Germany) according to the manufacturer’s protocol including an on-column DNAse treatment step. RNA purity and quality were evaluated on a NanoDrop1000 (Thermo Fisher Scientific Inc., Waltham, USA) and Agilent 2100 Bioanalyzer (Agilent Technologies, Santa Clara, USA). One hundred fifty nanograms of RNA of each sample was amplified and labeled using the Agilent “Low Input Quick Amp Labeling Kit” for One-Color Microarray-Based Gene Expression Analysis. In brief, the input RNA was reverse transcribed into cDNA, using an oligo-dT/T7-promoter hybrid primer which introduces a T7 promoter region into the newly synthesized cDNA. The subsequent in vitro transcription used T7 RNA polymerase, which simultaneously amplified the target material and incorporated cyanine 3-labeled CTP. cDNA synthesis and in vitro transcription were performed at 40 °C for 2 h, respectively. The labeled cRNA was purified using the Qiagen RNeasy Mini Kit and quantified on a NanoDrop1000. Finally, 1650 ng cRNA of each sample was hybridized at 65 °C for 17 h and the arrays subsequently scanned on an Agilent C-type Microarray Scanner G2565CA.

### Microarray data analysis

The microarray raw data were processed using the Agilent Feature Extraction Software (v10.7). A quantile between array normalization was applied, and differential expression was determined using the Bayesian adjusted *t*-statistic linear model of the “Linear Models for Microarray Data” (LIMMA) [21] package in R. The *p* values were corrected for a false discovery rate (Benjamini and Hochberg 1995) of 5%, and the gene expression levels were presented as log2-transformed intensity values. Normalized gene expression data have been deposited in NCBI’s Gene Expression Omnibus (GEO) and are accessible through GEO Series accession number GSE143408. Probes significantly different between the two groups were identified with an adjusted *p* value of ≤ 0.05 and an average absolute fold change of ≥ 1.5. For functional annotation and gene network analysis, filtered gene lists were examined using QIAGEN’s Ingenuity® Pathway Analysis (IPA®, QIAGEN, Redwood City, www.qiagen.com/ingenuity) and “Gene Set Variation Analysis” (GSVA) [22], “Gene Set Enrichment Analysis” (GSEA) [23], “Gene Ontology enRIchment anaLysis and visuaLizAtion tool” (GOrilla) [24], and GOsummaries [25].

### Comparative gene signature scoring

Gene sets of indicated signatures were acquired from Kyoto Encyclopedia of Genes and Genomes (KEGG), Gene Ontology, Ingenuity Pathway Analysis, REACTOME, and the Molecular Signature Database (hallmark gene sets, Broad Institute). GEO-deposited RNAseq data sets GSE104935 [26], GSE88752 [27], and GSE48403 [28] were downloaded as raw counts and processed by an edgeR pipeline with TMM normalization to obtain fragments per kilobase of transcript (fpkm) values. Mean expression was used to collapse multiple isoforms. Microarray data of this study were processed through limma pipeline, and Ensembl v77 probes were collapsed to gene level using mean log2 intensities. GSVA [22] was used for signature scoring, and non-scaled bubble plots were created with Morpheus webtool, with color indicating the direction of change of the GSVA scores (red = increased scores/gene sets increase in overall expression, blue = decreased scores/gene sets decrease in overall expression).

### Quantitative single cell analysis (qSCI) of lipid content by fluorescent microscopy

Prior to seeding, 96-well Ibidi optical plates were coated with 150 μl poly-l-ornithine (PLO, Sigma) and washed with PBS to increase cell attachment. PCa cell lines pre-treated with either 5% FBS + enzalutamide (10 μM) or 5% CSS were harvested three days prior completion of the indicated treatment times by trypsinization and seeded into PLO-coated 96-well Ibidi optical plates at a density of 6000 cells/well in their corresponding types of media (RPMI medium supplemented with either 5% FBS (D0), 5%FBS + enzalutamide (10 μM), or 5% CSS). After 3 days, the growth medium was removed, and the cells were washed with PBS and fixed with 4% paraformaldehyde (PFA). Lipids were stained with 0.25 μg/ml Nile Red (Sigma) overnight as described previously [29], and nuclear DNA was counterstained with 1 μg/ml 4′,6-diamidino-2-phenylindole (DAPI, Thermo Fisher). Alternatively, free cholesterol was stained with 50 μg/ml Filipin (Sigma), and DNA was counterstained with Draq5 (1 μM, Thermo Fisher). 500 cells/well were imaged using an InCell 2200 automated fluorescence microscope system (GE Healthcare Life Sciences). Quantitative analysis was performed with Cell Profiler Software [30] to measure total cellular content of neutral lipids, phospholipids, and free cholesterol based on mean fluorescence intensity and morphometric analysis of lipid droplets.

### qSCI of lipid transporter expression by immunofluorescence microscopy

Seventy-two hours after seeding of ATT pre-treated LNCaP cells in PLO-coated 96-well Ibidi optical plates and PFA fixation as described above, cells were permeabilized with TBS-0.1% Triton X-100 in PBS for 5 min followed by a 10 min treatment with TBS-2% BSA to block non-specific binding. Primary antibodies (LDLR (Abcam, ab52818) and SCARB1 (Abcam, ab217318)) were added at a 1:100 dilution in TBS-2% BSA (60 μl/well). After 24 h, the primary antibody was removed and cells were washed with TBS-0.1% Triton X-100 3 times for 5 min each. Secondary antibody (Alexa Fluor 568, Thermo Fisher) was added to cells at a 1:250 dilution in TBS-2% BSA (60 μl/well). After 1 h in the dark, the secondary antibody was removed and cells were washed with TBS-0.1% Triton X-100 3 times for 5 min each. DNA and F-actin were counterstained for 20 min in the dark with 1 μl/ml 4′,6-diamidino-2-phenylindole (DAPI, Invitrogen) and Alexa Fluor 647 phalloidin (Thermo Fisher), respectively. Staining solution was replaced with 200 μl PBS. Imaging and quantitative analysis were carried out as described above using the InCell 2200 System and CellProfiler software.

### qSCI of metabolic processes

ATT pre-treated cells were seeded as described above. For quantifying C16:0 fatty acid uptake, growth media was exchanged with 65 μl/well of serum-free RPMI media (Thermo Fisher) supplemented with 0.2% BSA (lipid-free, Sigma) and C16:0-Bodipy (5 μM, Thermo Fisher) and Mitotracker Orange CMTMRos (0.4 μM, Thermo Fisher) and incubated at 37 °C for 1 h. For quantifying cholesterol uptake, the growth medium was exchanged with 65 μl/well of serum-free RPMI media (0.2% lipid-free BSA) supplemented with 15 μM NBD (22-(N-(7-Nitrobenz-2-Oxa-1,3-Diazol-4-yl) Amino-23,24-Bisnor-5-Cholen-3β-Ol, Thermo Fisher) and incubated at 37 °C for 2 h. For quantifying lipoprotein complex uptake, the growth medium was exchanged with 65 μl/well of serum-free RPMI media (0.2% lipid-free BSA) supplemented with 1,1′-Dioctadecyl-3,3,3′,3′-Tetramethylindocarbocyanine (DiI)-labeled acetylated-LDL (15 μg/ml, Thermo Fischer) or DiI-labeled LDL (15 μg/ml, Thermo Fisher) and incubated at 37 °C for 2 h. Phosphatidylethanolamine uptake was measured as described above in serum-free RPMI media (0.2% lipid-free BSA) or, where noted, in conditioned media by incubation of cells at 37 °C for 1 h with NBD-PE (5 μM, 22-(N-(7-Nitrobenz-2-Oxa-1,3-Diazol-4-yl)-1, 2-Dihexadecanoyl-*sn*-Glycero-3-Phosphatidylethanolamine) triethylammonium salt, Thermo Fisher). After incubation, cells were fixed with 4% PFA. Cellular DNA and F-actin were then counterstained with DAPI and Alexa Fluor 647 Phalloidin (Thermo Fisher). Image acquisition and quantitative analysis were performed as described above.

For measuring cellular uptake of lyso-PC-A2, cells were seeded and processed as described above and incubated for 60 min at 37 °C with 65 μl/well of conditioned media supplemented with 5 μM Red/Green BODIPY® PC-A2 (A10072, 1-O-(6-BODIPY® 558/568-aminohexyl)-2-BODIPY® FL C5-sn-glycero-3-phosphocholine, Thermo Fisher). This phospholipid derivative carries in close proximity a red and green Bodipy fluorophore on the sn-1 and sn-2 acyl chains respectively that provides a capacity for dual emission fluorescence ratio detection. Cleavage of the BODIPY® FL pentanoic acid substituent at the sn-2 position by type 2 phospholipases results in decreased quenching by fluorescence resonance energy transfer (FRET) to the BODIPY® 558/568 dye attached to the sn-1 position. The result is a lipase-dependent increase in BODIPY® FL fluorescence emission detected in the range 515–545 nm (green). The FRET-sensitized BODIPY® 558/568 fluorescence signal is expected to show a reciprocal decrease. Notably, dose titration experiments with unconjugated BODIPY® FL pentanoic acid (Thermo Fisher) suggested a passive and slow route of cellular uptake when compared to longer chain FAs (C12:0 and C16:0, data not shown). Following the incubation, cells were fixed with 4% PFA, and cellular DNA and F-actin were then counterstained with DAPI and Alexa Fluor 647 Phalloidin (Thermo Fisher). Image acquisition and quantitative analysis were performed as described above. Lyso-PC-A2 uptake was calculated as ratio of green (cleaved lyso-PC-A2) vs red (uncleaved PC-A2) signal.

Membrane fluidity (differences in membrane lipid packaging) was measured by live ratio-fluoresce microscopy of ATT pre-treated cells after incubation in 80 μl/well of DPBS supplemented with 1 μM di-4-ANEPPDHQ (D36802, Thermo Fisher) for 30 min at 37 °C. DNA was counterstained with Hoechst 33342 (1 μg/ml, Sigma-Aldrich), and cells were washed once with DPBS. di-4-ANEPPDHQ-labeled cells (*n* > 750 cells, 8 wells/sample, 2 biological replicates) were imaged at 560 nm and 650 nm using the InCell 2200 System, where a spectral red shift is indicative of an increase in the disordered/liquid phase of the plasma membrane. Quantitative image analysis and calculation of the 560 nm/650 nm ratio was carried out with CellProfiler software.

Lipid peroxidation was measured by live ratiometric fluorescence microscopy of ATT pre-treated cells after incubation in 65 μl/well of serum-free RPMI media (0.2% lipid-free BSA) supplemented with 5 μM with BODIPY 581/591 C11 (Thermo Fisher) for 60 min at 37 °C. Peroxidation of the polyunsaturated butadienyl portion of the fluorescent dye results in a spectral fluorescence emission shift from orange (~ 590 nm) to green (~ 510 nm).

### Measurement of cell confluence and qSCI of cell death

Cell proliferation as a function of increasing cell confluence was measured by live imaging microscopy with the IncuCyte FLR and Zoom system (Essen BioScience, Ann Arbor, MI, USA). Parental or ATT pre-treated PCa cells were seeded as described above in 96-well Essen ImageLockTM plates (Essen BioScience, Ann Arbor, MI, USA). Images were acquired at 2-h intervals with a × 10 objective for up to 7 days.

For assessment of cell death, cells were seeded in 96 well plates (*n* = 3 wells/treatment with > 4000 cells/wells) as described above and treated with indicated compounds. At the end of the treatment period, cells were co-stained with Hoechst 3342 (1 μg/ml, total cell count) and propidium iodide (5 μg/ml, Sigma-Aldrich, dead cells). Cells were imaged using the InCell 2200 System, the images were analyzed with Cell Profiler software (Broad Institute) for total and dead cell count, and the percentage of dead cells was calculated based on the ratio of dead and total cells.

### Lipidome analysis

All extractions were performed in 2 ml glass vials. Methanol (220 μL) was added to cell pellet of approximately 2 million cells and vortexed. Internal standard (40 μl SPLASH Lipid-o-mix deuterated internal standard obtained from Avanti Polar Lipids, Alabaster, AL, and 20 μl n-Nonadecanoic acid (C19:0)) was added and briefly vortexed. Seven hundred seventy microliters of MTBE was added, and the mixture was incubated for 1 h at room temperature in a shaker. Phase separation was induced by adding 200 μl NH_4_CH_3_CO_2_ (150 mM). After vortexing for 20 s, the sample was centrifuged at 20,000*g* for 5 min. The upper (organic) phase was collected and stored at − 80 °C, then diluted into 2:1 MeOH to CHCl_3_ with 7.5 mM NH_4_CH_3_CO_2_ for mass spectrometry analysis.

Tandem mass spectrometry (MS) of the intact lipids was performed using a triple quadrupole mass spectrometer (6500 QTRAP, SCIEX, ON, Canada). The lipid extracts (as described above) were diluted 40-fold prior to analysis. Samples were directly infused into the electrospray ionization source of the mass spectrometer using a loop injection method, where 100 μl of sample was loaded into a sample loop using an autosampler and subsequently infused into the mass spectrometer by electrospray ionization at a flow rate of 20 μl/min. Lipid classes were targeted using either precursor ion or neutral loss scans. For quantification, SPLASH was added to cells prior to lipid extraction. Tandem MS data was processed using LipidView (version 1.3beta; SCIEX) using predefined target lists.

Hydrolysis and derivatization of lipids extracts to fatty acid methyl esters (FAME) was performed on-line using trimethylsulfonium hydroxide [31]. FAMEs were analyzed with a gas chromatograph coupled to a mass spectrometer (GC/MS – TQ8040; Shimadzu, Kyoto, Japan). The separation was carried out on a RTX-2330 capillary column (cyanopropyl stationary phase, 60 m × 0.25 mm, film thickness 0.20 μM; Restek, Bellefonte, PA, USA), and the electron ionization energy was set at 70 eV. Conditions for the analysis of FAMEs were as follows: carrier gas, He: column flow at 1 ml/min; 22:1 split ratio, injection volume 1 μl; injector temperature 240 °C; interface and ion source temperature 260 C. GC oven temperature was maintained at 100 °C for 1 min, thermal gradient 100 to 140 °C at 10 °C/min, 140 to 175 °C at 6 °C/min, and 175 to 200 °C at 10 °C/min and hold for 1 min, followed by 200 to 250 °C at 5 °C/min and hold for 4 min. The data were acquired with Q3 scan mode from *m/z* 50–650. For data collection, the MS spectra were recorded from 4.6 to 28.33 min. The data was processed in GC/MS solution software (Shimadzy, Kyoto, Japan). Fatty acids were identified based on retention time alignment with reference FAMEs from a mixture of standards (Food Industry FAME mix (Table S6), Restek, Bellefonte, PA, USA). All samples were normalized to internal standard C19:0, and peak areas of relevant FAMEs were subtracted from the negative control samples.

### ^13^C carbon tracing of metabolites by mass spectrometry

LNCaP cells were treated with DMSO or enzalutamide (10 μM) for up to 21 days as described above. Seventy-two hours prior to harvesting, media was replaced with complete growth media supplemented with uniformly labeled ^13^C-acetate (500 μM, Sigma-Aldrich). Metabolites were extracted using 80% methanol in water extraction buffer (Sigma) that was supplemented with uniformly deuterated myristic acid (2 μM, Sigma) as an internal control. Samples were normalized based on total protein measurements in precipitates by bicinchoninic acid assay (BCA, Pierce).

GC/MS analyses were performed using an Agilent 7890A GC equipped with a DB-35MS (30 m, 0.25 mm i.d., 0.25 μm) capillary column (Agilent Technologies), interfaced with a triple quadruple tandem mass spectrometer (Agilent 7000B, Agilent Technologies) operating under ionization by electron impact at 70 eV. The injection port, interface, and ion source temperatures were maintained at 230 °C. The temperature of the quadrupoles was maintained at 150 °C. The injection volume was 1 μl, and samples were injected at a 1:25 split ratio. Helium flow was kept constant at 1 ml/min.

GC oven temperature was maintained at 60 °C for 1 min, increased to 300 °C at 10.0 °C/min. Post-run temperature was 325 °C, kept for 5 min. The mass spectrometer operated in SIM mode and cholesterol-tms derivative was determined from the fragment at m/z 458,4 (C30H54OSi) as M+0; also, m/z fragments representing M+1 up to M+16 were detected in order to determine the ^13^C-acetate incorporation into cholesterol.

### Statistical analysis

Statistical analysis was performed with GraphPad Prism 8.3 (GraphPad Software, San Diego, CA) and R Studio (RStudio, Boston, MA). Data reported and statistical tests are described in the figure legends.

## Results

### Therapy-induced remodeling of metabolic networks leads to cellular quiescence and multidrug tolerance

**Fig. 1:**
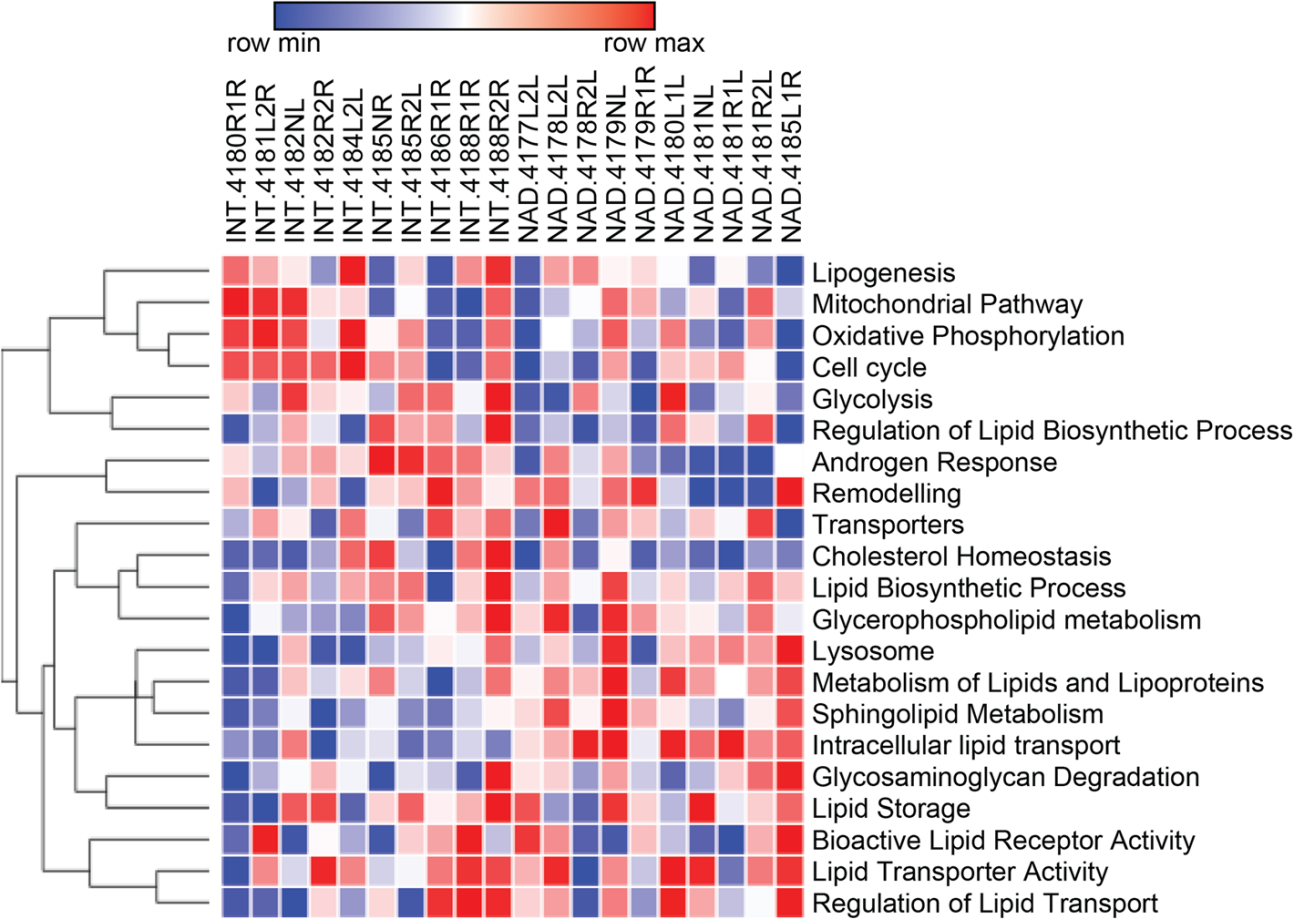
Therapy-induced reprograming of metabolic networks in a longitudinal PCa tumor xenograft model of CRPC progression. **a** Gene set variation analysis (GSVA) of ten tumors at PSA nadir (NAD) compared to ten tumors from sham-castrated mice (INT, intact) was generated based on previously published microarray analysis of LNCaP xenograft tumors resected at different time points after castration [32]. Heatmaps were generated with a hierarchical clustering algorithm using completed linkage and Euclidean distance measures and scaled by *z* score (red = positive *z* score, blue = negative *z* score)

Our previous longitudinal analysis of LNCaP tumor xenografts in a model of CRPC progression following castration showed that CRPC tumors contained significantly higher lipid levels than tumors resected from sham-castrated mice [32]. These lipids included essential fatty acid (FA) derivatives, indicating that development of CRPC is associated with enhanced uptake of exogenous, diet-derived lipids [16]. A detailed review of transcriptomics data from this study by gene set variation analysis (GSVA) revealed that castration caused a negative enrichment of pathways involved in lipogenesis, mitochondrial activity, and oxidative phosphorylation in tumors at nadir of serum PSA levels compared to non-castrated tumor-bearing mice (intact, control). In support of therapy-induced changes to tumor lipid supply, tumors at PSA nadir showed increased enrichment in pathways of lipoprotein metabolism, lipid storage, lipid transporter, and lysosomal activity (Fig. 1). The complete longitudinal analysis including that from regressing and recurrent tumor samples is shown in Fig. S1A, highlighting the dynamic changes in response to androgen-targeted therapies (ATTs) during progression to CRPC.

**Fig. 2:**
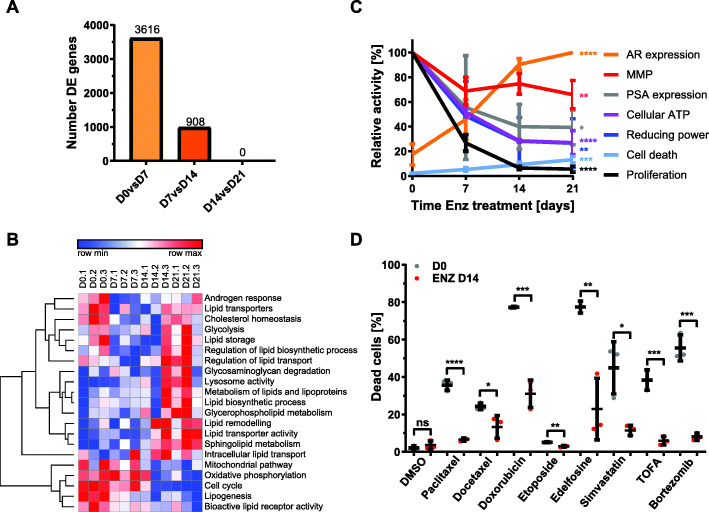
Therapy-induced metabolic reprogramming of LNCaP PCa cells is associated with cellular quiescence and multi drug tolerance. **a** LNCaP cells were treated for 0, 7, 14, and 21 days with AR antagonist enzalutamide (Enz, 10 μM), and samples were analyzed by microarray analysis. The number of differentially expressed genes between indicated pairwise comparisons are shown (FDR 1.5fold, *p* < 0.05). **b** Gene set enrichment analysis of the microarray data described in **a** for the analysis of indicated pathways. **c** LNCaP cells were treated as in **a**, and samples were analyzed by qRT-PCR (AR and PSA mRNA expression), live-cell confluence imaging (proliferation), CellTiter-Glo assay (cellular ATP levels), PrestoBlue assay (cellular reducing power), quantitative single-cell imaging (qSCI) of MitoTracker Orange CMTMRos (mitochondrial membrane potential, MMP), and dead/live staining (cell death) for functional characterization of Enz-induced metabolic changes (*n* = 3, activities relative to day 0 with the exception of AR levels, where levels were calculated relative to day 21). **d** LNCaP cells were cultured in growth media without (D0) or with Enz (10 μM, ENZ D14) for 14 days including the final 24 h in the presence of vehicle control (DMSO) or the indicated compounds (paclitaxel 2.5 μM, docetaxel 2.5 μM, doxorubicin 2.5 μM, etoposide 5 μM, edelfosine 7.5 μM, simvastatin 30 μM, TOFA 60 μM, bortezomib 2.5 μM). The percentage of dead cells was calculated based on qSCI of Hoechst 3342 (total cell count) and propidium iodide (dead cells) staining (*n* = 3 wells/treatment with > 4000 cells/well; ns, not significant, *p < 0.05, **p < 0.01, ***p < 0.001, ****p < 0.0001, one-way ANOVA followed by Dunnett’s multiple comparisons test compared to D0, representative result of 2 independent experiments)

To investigate therapy-induced longitudinal changes to lipid metabolism on the functional and molecular level, the AR-positive, androgen-dependent LNCaP PCa cell line was grown for up to 21 days in the presence of enzalutamide (Enz) to block AR function. Pairwise sequential comparisons of transcriptome data generated from samples taken prior (day 0) and 7, 14, and 21 days post commencement of Enz treatment showed that LNCaP cells reached transcriptional stasis after 14 days of Enz, i.e., the set of 4524 differentially expressed genes (D0 vs D14) remained unchanged when compared to 21 days Enz (D0 vs D21, Fig. 2a). This suggested that the phenotype induced by Enz was transcriptionally established by day 14 of Enz treatment and persisted for another week. This longitudinal analysis further highlighted that Enz treatment periods beyond prevailing protocols to assess the acute response to ATTs in vitro (2-4 days) [16] identified an additional 908 (25%) differentially expressed genes.

GSVA yielded enrichment scores that clustered pathways into three types of adaptive responses to Enz treatment when compared to control (day 0): pathways that changed to and persisted as either negatively or positively enriched and dynamic pathways that, after temporarily displaying negative enrichment (days 7 and 14), reverted to a positive enrichment seen prior treatment commencement. GSVA revealed negative enrichment for known androgen-activated pathways, e.g., lipogenesis, cell cycle, and mitochondrial pathways, including oxidative phosphorylation (Fig. 2b). Pathways with positive enrichment were associated with lysosomal function, lipid metabolism, lipid remodeling, and extra- and intracellular lipid transport (Fig 2b). Dynamic pathways with transient negative enrichment that reverted to their initial positive enrichment seen prior the start of treatment were associated with the androgen response, lipid transporters, cholesterol homeostasis, glycolysis, lipid storage, and regulation of lipid biosynthesis and lipid transport (Fig. 2b). GSVA suggested a substantial reconfiguration of the lipid metabolism network in response to Enz over the treatment period of 21 days and were consistent with the in vivo results (Fig. 1). Consistent with this, protein mass spectrometry of LNCaP cells treated for 0 and 21 days with Enz and gene ontology analysis revealed enrichment of membrane lipid metabolic processes, glycolipid metabolism, and lysosome function (Fig. S1B and Table S2).

Extended cell culture (several months) in androgen-depleted or AR-antagonized conditions ultimately yielded androgen-independent or Enz-resistant CRPC cells that reinitiated proliferation [6, 8, 33, 34]. Our comparative signature scoring of transcriptomics data sets of relevant studies performed in cell culture leading to Enz resistance in LNCaP and C4-2B cells [26], in castrated mice progressing to primary and secondary CRPC after serial tumor xenograft transplantation of LAPC9 and LNCaP cells [27], and matching PCa tumors from patients before and after androgen deprivation therapy for 22 weeks [28] by GSVA using lipid metabolism-related gene sets showed extensive concordance between cell culture models, pre-clinical tumor xenograft models, and clinical samples of ATT resistance (Fig. S1C). This analysis further highlighted the dynamic nature of some therapy-induced metabolic changes, potentially limiting their value as co-treatment options based on the therapeutic window.

The observed time-dependent increase in AR mRNA expression by Enz over 21 days (Fig 2c, Fig. S2A) was consistent with reported resistance mechanisms to ATT [2, 4]. mRNA levels of *PSA*/*KLK3* remained decreased (Fig. 2c) and a large subset of other AR-activated and suppressed genes (Fig. S2B) did not change, suggesting that AR activity remained suppressed. Consistent with the de-differentiation effect of AR-antagonism, transcriptional levels of several stemness markers were significantly increased (Table S1) [35]. Furthermore, Enz-treated LNCaP cells showed a time-dependent strong reduction in cellular reducing power (− 70%), ATP levels (− 60%), and mitochondrial membrane potential (− 30%), which stabilized after 14 days (Fig. 2c, Fig. S2C). Proliferation almost completely ceased after 14 days of Enz treatment, but cell death was only modestly increased (Fig. 2c, Fig. S2D) which was possibly due to protection via significantly increased autophagy (Fig. S2E). This low cycling phenotype was underpinned by a strong downregulation of genes associated with cell cycle progression (Fig. 2b). Enz-treated LNCaP cells adopted a spindle-like cell morphology marked by a reduced cell size, increased cell perimeter, and major axis length (Fig. S2F). Transcriptionally, metabolically, and proliferation-wise, LNCaP cells responded to extended Enz treatment by entering into a state of cellular quiescence. Associated with this phenotype was the acquisition of multi-drug tolerance (MDT) to a range of lipogenesis inhibitors and clinically relevant chemotherapeutics for CRPC in LNCaP, C4-2B, and DuCaP cells (Fig. 2d and S2G), indicating that this adaptive response to Enz is shared across multiple AR-positive PCa cell lines. Despite MDT, a limited drug screen revealed enhanced sensitivity of PCa cells in the Enz-tolerant state to U18666A (Fig. S2H), an inhibitor of the lysosomal Niemann-Pick C1 protein (NPC1). NPC1 together with NPC2 are critical for lysosome to cytoplasm transport of exogenous cholesterol [36], and both proteins show strongly increased expression in localized and metastatic PCa (Fig. S2H). Notably, culturing of LNCaP cells in androgen-depleted growth media (CSS) for 14 days showed similar adaptive responses when compared to Enz, e.g., cellular ATP levels and reducing power, proliferation, and MDT (Figs. S2C, S2D, and S2G), suggesting that both ATTs cause similar metabolic reprograming that leads to the persister cell state.

### Androgen-targeted therapy increased cellular lipid content

**Fig. 3:**
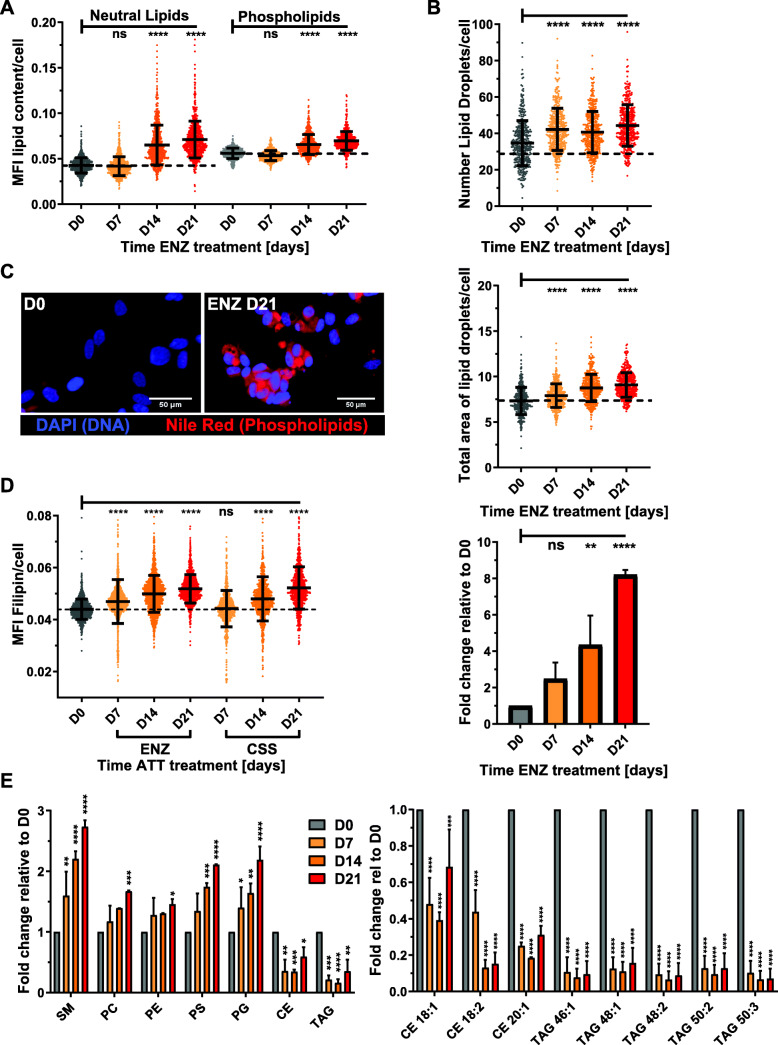
Enzalutamide increased cellular lipid content. LNCaP cells were treated for the indicated times with Enz (10 μM) and analyzed by qSCI based on the mean fluorescence intensity (MFI) of Nile Red to measure **a** cellular neutral lipid and phospholipid content and **b** number of lipid droplets (LDs)/cell and total area of LDs/cell (*n* > 9000 cells/treatment, mean ± SD, representative result of 3 independent experiments). Perilipin mRNA expression was measured by qRT-PCR (*n* = 3, mean ± SD). **c** Representative images of D0 and D21 showing phospholipid and DNA staining as described in **a**. **d** LNCaP cells were treated as in **a** or grown in androgen-depleted media (CSS, charcoal-stripped serum) for the indicated times, stained with Filipin, and free cholesterol was measured by qSCI (*n* > 9000 cells/treatment, mean ± SD, representative result from 2 independent experiments). **e** LC/MS shotgun lipidomics of intact lipid species of LNCaP cells treated as described in **a**. Total combined sum compositions of indicated lipid classes (left panel, SM, sphingomyelin; PC, phosphatidylcholine; PE, phosphatidylethanolamine; PS, phosphatidylserine; PG, phosphatidylglycerol; CE, cholesterol ester; TAG, triacylglycerol), and individual CE and TAG species (right panel) are reported as fold change relative to D0 (*n* = 2, mean ± SD). Statistical analysis: **a**–**d** one-way ANOVA followed by Dunnett’s multiple comparisons test compared to D0; **e** two-way ANOVA followed by Tukey’s multiple comparisons test relative to D0; ns, not significant, *p < 0.05, **p < 0.01, ***p < 0.001, ****p < 0.0001

The above results suggested major changes to lipid metabolism networks by Enz. Previous studies, including our own work, have demonstrated that androgens strongly enhance cellular lipid content of PCa cells through enhanced lipogenesis [37, 38] and lipid uptake [16] and that AR antagonists block these lipid supply pathways. To measure Enz-induced changes to the cellular content of neutral lipids, lipid droplets, phospholipids, and free cholesterol, we used quantitative single-cell imaging (qSCI) of two fluorescent lipophilic dyes, Nile Red and Filipin. Nile Red is able to distinguish between phospholipids and neutral lipids [e.g., triacylglycerols (TAGs) and cholesteryl esters (CEs) stored in lipid droplets] based on their difference in hydrophobicity having separate fluorescence emission maxima [39]. Filipin is a selective fluorescent stain for free, non-esterified cholesterol which is mainly found in the plasma membrane [40]. Contrary to the expected lipid depletion resulting from loss of androgen-enhanced lipid supply, qSCI of Nile Red-stained LNCaP cells demonstrated that Enz significantly increased neutral lipid and phospholipid content with increasing time of treatment (Fig. 3a–c). Morphometric analysis of lipid droplets (LDs) showed that the LD number and average total area of LDs per cell was also significantly increased with treatment time (Fig. 3b top and middle panel). Consistent with this, the mRNA expression of the LD surface protein PLIN1 was significantly increased by Enz treatment (Fig. 3b bottom panel). qSCI of Filipin-stained LNCaP cells showed that Enz induced a significant increase in free cholesterol content (Fig. 3d). Similar observations were made when LNCaP cells were maintained in androgen-depleted media (CSS, data not shown).

For molecular analysis of the Enz-induced longitudinal changes to the lipidome, we next ran electrospray ionization mass spectrometry (ESI-MS) of lipidomics of lipid extracts prepared from LNCaP cells treated with vehicle control (FBS) or for 7, 14, and 21 days with Enz. Principal component analysis showed that each time point sample group of the Enz-treatments clustered distinctly from the vehicle treated control group (FBS) and to a lesser extent from each other (Fig. S3A). LNCaP cells significantly increased cellular levels of sphingomyelin (SM), phosphatidylcholine (PC), phosphatidylethanolamine (PE), phosphatidylserine (PS), and phosphatidylglycerol (PG) over the time course of treatment (Fig. 3e left panel, Fig. S3B-F). While all major membrane lipid classes were increased by Enz, neutral lipids like CEs and TAGs stored within LDs were significantly decreased (Fig. 3e right panel, Figs. S3G and S3H). Of the top 50 deregulated lipid species measured, 86% of these were found to have increased abundance with increasing time of Enz treatment (Fig. S3I). Notably, the majority of these were PC and PE phospholipids that make up ~ 70% of the lipid content of mammalian cells [41], providing further evidence that Enz increased cellular lipid content. Together, qSCI and lipidomics analyses demonstrated that Enz induced substantial remodeling of all major lipid classes, leading to a net increase in cellular lipid content.

### Enhanced lipid uptake fuels cellular lipid accumulation

**Fig. 4:**
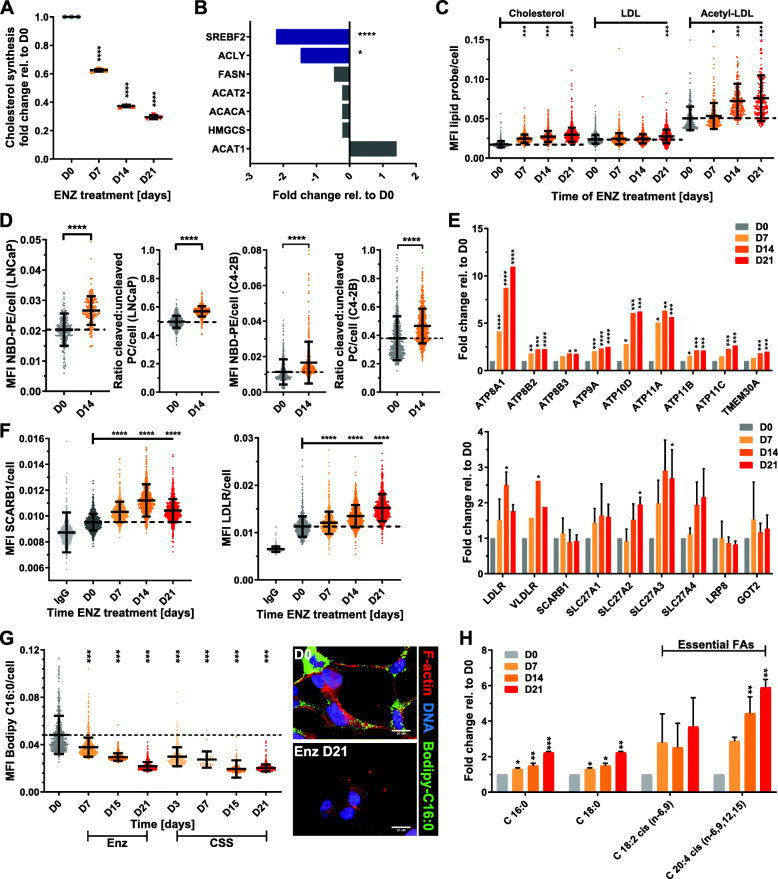
Increased cellular lipid levels are fueled by enhanced lipid uptake. **a** LNCaP cells were treated for the indicated times with Enz (10 μM) including the final 72 h in the presence of ^13^C-acetate. Incorporation of ^13^C into cholesterol via lipogenesis was measured by GC/MS (*n* = 3, mean ± SD). **b** Protein (blue) and mRNA (gray) expression of indicated enzymes was measured by quantitative proteomics with isobaric mass tag labels and microarray analysis, respectively (*n* = 3, mean ± SD). **c** LNCaP cells were treated as described in **a**, and cellular uptake of indicated fluorescent lipid probes was measured by qSCI based on the mean fluorescence intensity (MFI) of NBD-cholesterol, LDL(DiL), acetylated-LDL(DiL), or **d** NBD-PE and PC-A2 in LNCaP and C4-2B cells (*n* > 9000 cells, mean ± SD, representative of 2 independent experiments). **e** Fold-change mRNA expression changes of indicated lipid transporters relative to D0 of Enz-treatment were calculated from the microarray data described in Fig. 2a (top panel) or measured by qRT-PCR (bottom panel). **f** LNCaP cells were treated for the indicated times with Enz (10 μM), and protein expression of lipid transporters SCARB1 (left) and LDLR (right) was measured using immunofluorescence microscopy (IgG = antibody control, *n* > 9000 cells, mean ± SD, representative result from 2 independent experiments). **g** LNCaP cells were treated with Enz (10 μM) or grown in androgen-depleted media (CSS, charcoal-stripped serum) for the indicated times, and FA uptake was measured by qSCI based on the MFI of cellular Bodipy-C16:0 (*n* > 9000 cells, mean ± SD, representative result from 3 independent experiments). **h** LNCaP cells were treated for the indicated times with Enz (10 μM), and fatty acid content was analyzed by GC/MS FAME (*n* = 2, mean ± SD). Statistical analysis: **a**–**h** one-way ANOVA followed by Dunnett’s multiple comparisons test compared to D0; *p < 0.05, **p < 0.01, ***p < 0.001, ****p < 0.0001

As shown above, therapy-induced persister cells displayed multidrug tolerance to lipogenesis inhibitors (Fig. 2d), suggesting that de novo synthesis played a limited role in cellular lipid accumulation. Indeed, metabolic tracing of ^13^C-acetate by mass spectrometry revealed that de novo cholesterol synthesis decreased by 71% (*p* > 0.0001) after Enz treatment of LNCaP cells for 21 days when compared to control (Fig. 4a). No significant incorporation of ^13^C-glucose, another key carbon source for lipogenesis, into lipids was measured (data not shown), and already low basal glucose uptake was significantly reduced by Enz (Fig. S4A). Consistent with this, transcriptomics and proteomics analysis showed that key enzymes of de novo cholesterol and FA synthesis pathways were significantly downregulated by Enz treatment (Fig. 4b, Table S1), suggesting that alternative lipid supply pathways were responsible for therapy-induced lipid accumulation.

To measure cellular lipid uptake, qSCI with various fluorophore labeled lipid probes (NBD-cholesterol, DiI-LDL, DiI-acetylated LDL, Bodipy-C16:0, NBD-phosphatidylethanolamine, Bodipy-phosphatidylcholine) was conducted. Enz treatment significantly increased cellular uptake of free NBD-cholesterol and DiI-complexed low-density lipoprotein (LDL) and acetylated LDL in LNCaP cells, as well as NBD-phosphatidylethanolamine and Bodipy-lyso-phosphatidylcholine in LNCaP and C4-2B cells (Fig. 4c, d) in a time-dependent manner. Consistent with increased lipid uptake and cellular content, Enz significantly increased the mRNA levels of several lipid transporters, including phospholipid transporters and co-factors belonging to the family of P4-ATPases/flippases (Fig. 4e, top panel; Table S1) and other cargo-selective lipid transporters, including low-density lipoprotein receptor (LDLR) (Fig. 4e bottom panel). Protein expression of several of these lipid transporters was upregulated by several orders of magnitude in clinical samples of bone metastatic CRPC when compared to localized, hormone-naïve PCa and normal prostate gland [42] (Fig. S4B), suggesting that enhanced lipid uptake might remain activated throughout the development of ATT resistance and CRPC.

LDL particles are a bulk supply of apolipoproteins and complex lipids, including CEs, TAGs, and phospholipids to cells via receptor-mediated endocytosis of LDLR and scavenger receptor SCARB1 [43, 44]. Consistent with increased cellular uptake of LDL and acetylated LDL, protein expression of lipid transporters LDLR and SCARB1 was significantly enhanced by Enz. (Fig. 4f). Surprisingly, uptake of saturated free FA (Bodipy-C16:0) was drastically decreased by both androgen-depleted serum (CSS) and Enz treatment over time (Fig. 4g), suggesting that ATT-induced changes to transporter-mediated lipid uptake are specific. Gas chromatography-mass spectrometry (GC/MS) analysis of derivatized FAs released upon saponification of complex lipids revealed that saturated FAs (C16:0 and C18:0) and essential PUFAs linoleic acid (C18:2n-6) and its derivative arachidonic acid (C20:4n-6) were significantly increased by Enz with increasing time of treatment (Figs. 4h and S4C). Together, the above results suggested that saturated FA requirements from exogenous sources were preferably met by uptake of complex lipids (e.g., TAGs, CEs, phospholipids) which are also rich sources of PUFAs (Fig. S4D). Despite this observed cargo selectivity, Enz also enhanced cargo non-selective mechanisms involved in lipid scavenging, e.g., macropinocytosis [45] and tunneling nanotubes (TNTs, Figs. S4E and S4F) [46]. Together, these results demonstrated that Enz-treated PCa cells overall enhanced lipid uptake through cargo-selective and non-selective mechanisms.

### Therapy-induced lipid remodeling increases membrane fatty acid desaturation and fluidity

**Fig. 5:**
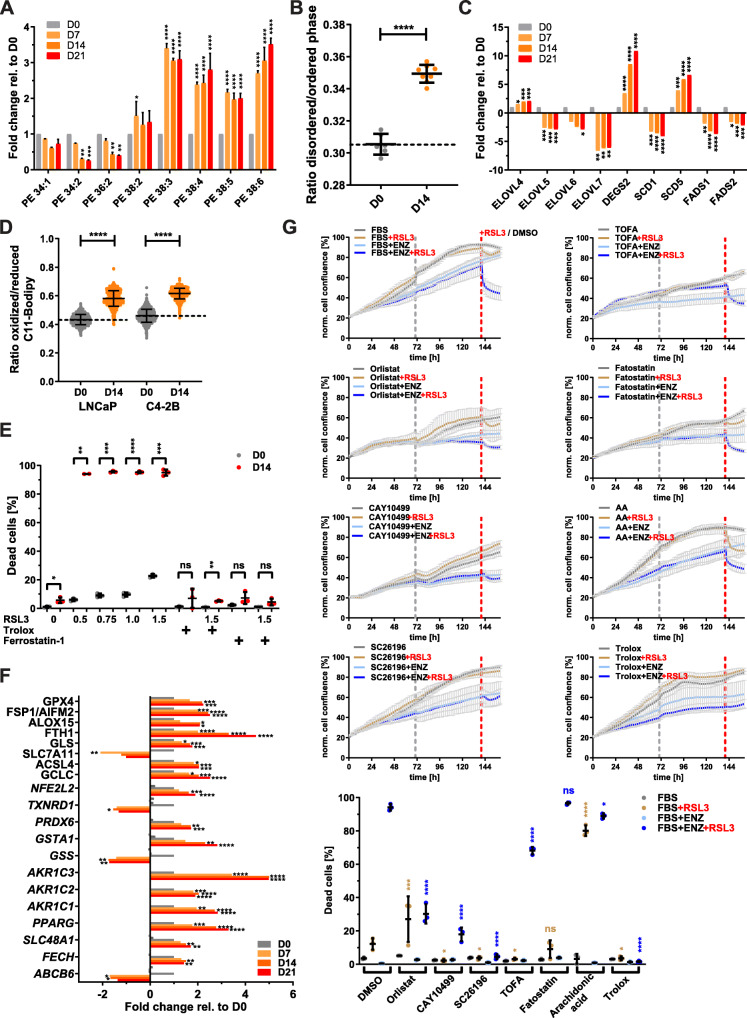
Therapy-induced lipid remodeling leads to increased membrane PUFA levels and lipid peroxidation and underpins GPX4 dependence. **a** LNCaP cells were treated for the indicated times with Enz (10 μM), and lipids were analyzed by LC/MS. Individual sum compositions of phosphatidylethanolamine are shown (*n* = 2, mean ± SD). **b** LNCaP cells were treated as in **a**, and membrane fluidity was measured by qSCI based on the mean fluorescence intensity (MFI) of Di-4-ANEPPDHQ (*n* = 8 wells, ~ 750 cells/well, mean ± SD, representative results from 2 independent experiments). **c** Relative mRNA expression changes of indicated lipid remodeling enzymes (elongases and desaturases) were calculated from the microarray data described in Fig. 2a. **d** LNCaP cells were treated for the indicated times with Enz (10 μM), and lipid peroxidation was measured by qSCI based on the mean fluorescence intensity (MFI) of Bodipy-C11 (*n* > 9000 cells, mean ± SD, representative results from 2 independent experiments). **e** LNCaP cells were cultured in growth media without (D0) or with Enz (10 μM, D14) for 14 days including the final 24 h in the presence of DMSO (0) or the indicated concentrations (0.5–1.5 μM) of GPX4 inhibitor RSL3. Lipid peroxide scavenger Trolox (100 μM) and ferroptosis inhibitor Ferrostatin-1 (10 μM) were used as controls to demonstrate cell death through ferroptosis. The percentage of dead cells was calculated based on qSCI of Hoechst 3342 (total cell count) and propidium iodide (dead cells) staining (*n* = 3 wells/treatment with > 4000 cells/well, representative result of 3 independent experiments). **f** Relative mRNA expression changes of indicated key factors of ferroptosis and glutathione homeostasis were calculated from the microarray data described in Fig. 2a. **g** LNCaP cells seeded in a 96-well plate were co-treated with the indicated compounds (orlistat 10 μM, CAY10499 17.5 μM, SC26196 30 μM, TOFA 2.5 μM, fatostatin 5 μM, arachidonic acid (AA) 20 μM, Trolox 100 μM) in the absence (FBS) or presence of Enz (10 μM, FBS+ENZ), and cell confluence was measured every 2 h by live cell imaging with an IncuCyte system (*n* = 3, mean ± SD). The gray dotted line indicates the time point of media change. After 7 days of co-treatment, cells were treated with vehicle control (DMSO/FBS) or RSL3 (1.25 μM) to assess GPX4 dependence by monitoring changes to cell confluence for another day. The red dotted line indicates the start of RSL3 treatment. Cell death was then measured by qSCI (bottom graph) as described in (**e**; *n* = 3 wells/treatment with > 4000 cells/well, representative result of 2 independent experiments). Statistical analysis: **a** two-way ANOVA followed by Tukey’s multiple comparisons test relative to D0; **b**–**f** one-way ANOVA followed by Dunnett’s multiple comparisons test compared to D0 or FBS+RSL3 (**g**, light brown labels) and FBS+ENZ+RSL3 (**g**, blue labels); ns, not significant, *p < 0.05, **p < 0.01, ***p < 0.001, ****p < 0.0001

The above results showed that Enz affected all major lipid classes and strongly increased cellular content of phospholipids, suggesting changes to membrane composition and function. Exemplified by lipid species of the phosphatidylethanolamine (PE) phospholipid class, PEs with shorter FA chains (34:2 and 36:2) were significantly decreased, while PEs with longer and more unsaturated FA chains (38:3, 38:4, 38:5, and 38:6) were significantly increased by Enz treatment compared to control (Fig. 5a). Similar trends were seen in SM, PC, PE, PS, and PG lipid classes (Fig. S3B-F), where an enrichment of phospholipids with long-chain PUFAs was observed. Integrated analyses of all dysregulated fatty acid species detected by GC/MS FAME analysis (Fig. S4C) or proteins detected by proteomics (Fig. S1B) with the mRNA expression changes by microarray (Fig. 2) of LNCaP cell treated with vehicle control or Enz for 21 days demonstrated significant enrichment of multiple pathways involved in PUFA metabolism (Fig. S5A), including those of essential PUFAs (e.g., linoleic acid and arachidonic acid).

Consistent with increased PUFA content, Enz-treated LNCaP cells displayed increased membrane fluidity (Fig. 5b). Regarding the mechanisms underlying the increase in desaturation and elongation of PUFAs, we noted a significant reduction of PUFA containing storage lipids (CEs and TAGs; Figs. S3G and S3H). Apart from enzymes that play primarily a role in the elongation of very long FAs (> 24 carbon atoms) and the desaturation of sphingolipids and mono-unsaturation of FAs (*ELOVL4* [47], *DEGS2* and *SCD5*), mRNA expression of most FA elongases and desaturases was significantly decreased by Enz (Fig. 5c. Table S1). In support of this, a review of microarray data from our LNCaP tumor xenograft model of CRPC progression [32] revealed that the expression of elongases and desaturases was overall reduced in regressing tumors and tumors at PSA nadir when compared to tumors from sham-castrated mice (Fig. S5B and S5C). Notably, this expression pattern reversed and increased in recurring tumors and CRPC when compared to tumors from sham-castrated mice. Furthermore, FA elongation and de novo FA synthesis share the requirement of malonyl-CoA as a C2 carbon donor, which is produced by acetyl-CoA carboxylase (ACACA). As shown in Fig. 4b, ACACA mRNA expression was reduced, providing further support that Enz-treated cells were increasingly acquiring PUFAs from uptake of serum-derived lipids (Fig. S4D) and by mobilizing PUFAs from lipid storage (e.g., from CEs and TAGs) with ongoing therapy (Figs. S3G and S3H).

### PUFA enrichment of membrane lipids is associated with increased lipid peroxidation and dependency on GPX4 activity

High PUFA content of membrane phospholipids renders cells susceptible to oxidative stress through iron-dependent lipid peroxidation. In contrast, PUFAs stored as CEs and TAGs within lipid droplets are protected [48]. Excess amounts of PEs containing oxidized arachidonic acid (C20:4) or adrenic acid (C22:4) have been shown to trigger ferroptosis, a form of regulated, non-apoptotic cell death [12]. As shown above, Enz increased cellular levels of arachidonic acid (Fig. 4h) and PEs esterified with PUFAs (Fig. 5a). qSCI with the lipid peroxidation sensor C11-Bodipy and ratiometric analysis revealed that treatment of LNCaP and C4-2B cells for 14 days with Enz significantly increased lipid peroxidation (Fig. 5d). Glutathione peroxidase (GPX4) is critical for the reduction of toxic lipid hydroperoxides to their corresponding non-deleterious alcohols at the expense of reduced glutathione and thereby prevents cellular damage and induction of ferroptosis. LNCaP, C4-2B, DuCaP, and 22RV1 cells treated with Enz or CSS displayed hypersensitivity to the GPX4 inhibitor RSL3 as indicated by significantly increased cell death (Fig. 5e and S5D). Thus, several AR-positive PCa cell lines demonstrated increased dependency on GPX4 activity in response to ATTs, a characteristic previously shown to be associated with drug-induced persister cells in other types of cancer in response to different therapies [9, 49]. Cytotoxicity of RSL3 was completely suppressed by antioxidant and lipid peroxide scavenger Trolox, a vitamin E derivative, and ferroptosis inhibitor Ferrostatin-1 (Figs. 5e and S5D), confirming ferroptosis as the major cell death mechanism. Enhanced GPX4 dependence was detected as early as 4–8 days after commencement of ATTs (Enz and ADT) in LNCaP and C4-2B cells (Fig. S5E) and was fully reverted when cells were given a therapy holiday for three or more days (data not shown). Consistent with altered GPX4 dependence, mRNA levels of several genes involved in lipid peroxidation, ferroptosis, and redox homeostasis, including GPX4 and the recently identified ferroptosis suppressor protein 1 (FSP1/AIFM2) [10, 11], were significantly altered by Enz (Fig. 5f). In addition, Nrf2 (*NFE2L2*) is a master regulator of the antioxidant response [50], and several of its target genes that regulate iron and glutathione metabolism were also upregulated by Enz (Fig. 5f). Gene ontology analysis of protein expression changes (Fig. S1B) showed significant enrichment of pathways related to oxidative stress and selenocysteine metabolism which is an amino acid critical for the antioxidant function of selenoproteins (e.g., glutathione peroxidases, thioredoxin reductases, iodothyronine deiodinases, Fig. S5F).

Next, we tested if the above processes associated with lipid remodeling are critical mechanisms that underpin the development of GPX4 dependence in PCa persister cells. Indeed, acquisition of ferroptosis hypersensitivity, as measured by RSL3-induced cell death, was significantly suppressed in LNCaP (Fig. 5g) and C4-2B cells (data not shown) when Enz was combined with non-toxic doses of inhibitors directed against lipase activity (Orlistat and CAY10499) [51] or FA delta-6 desaturation (SC26196) [52]. Co-inhibition of the critical lipogenesis transcription factor SREBF1 with fatostatin [53] or ACACA (acetyl-CoA to malonyl-CoA conversion) and SCD1 (FA desaturation) with dual inhibitor TOFA [54] showed no or only limited reduction in ferroptosis sensitivity (Fig. 5g). The high protective efficiency of broad spectrum lipase inhibitor CAY10499 and FADS2 desaturase inhibitor SC26196 against RSL3-induced cell death in ENZ-treated LNCaP cells was comparable to that of Trolox (Fig. 5g) and Ferrostatin-1 (data not shown), suggesting that the co-inhibited processes are fundamental for developing Enz-induced GPX4 dependence. Similar results were observed when LNCaP cells were cultured in CSS, though the effect and efficacy of some co-treatments were different when compared to direct inhibition of AR with Enz, including that of fatostatin (Fig. S5G), suggesting that changes to the media composition (e.g., free FA levels, Fig. S4D) by charcoal stripping might have confounding effects. In support of this, supplementation of growth media with the PUFA arachidonic acid strongly intensified RSL3 sensitivity in parental, non-ATT treated cells (Fig. 5g), whereas reduced serum concentrations were protective (data not shown). This suggested a functional link of PUFA uptake mechanisms with ferroptosis sensitivity. Thus, interference with lipid supply and lipid remodeling pathways that were altered by ATTs (Enz and ADT) efficiently suppressed the acquisition of ferroptosis hypersensitivity. This demonstrated that therapy-induced GPX4 dependence can be modulated by rationally informed co-treatments targeting lipid remodeling and by altering PUFA and antioxidant levels of serum.

## Discussion

Despite initial tumor regression following targeted cancer therapies, development of drug resistance and disease progression ultimately leading to patient death remain major clinical challenges of many pharmacological interventions in various types of cancer, including PCa. Previous studies of resistance to androgen receptor-targeted therapies (ATTs) in PCa analyzed models after extensive treatment periods (months to years) with fully developed resistance as indicated by reactivated cell proliferation [6, 8, 33, 34]. Different to these studies, we hypothesized that delineating early events of ATT-induced metabolic reprogramming might provide novel co-targets that could extend the efficacy of ATTs, thereby delaying therapy failure and disease progression to currently incurable CRPC and Enz-resistant CRPC.

**Fig. 6:**
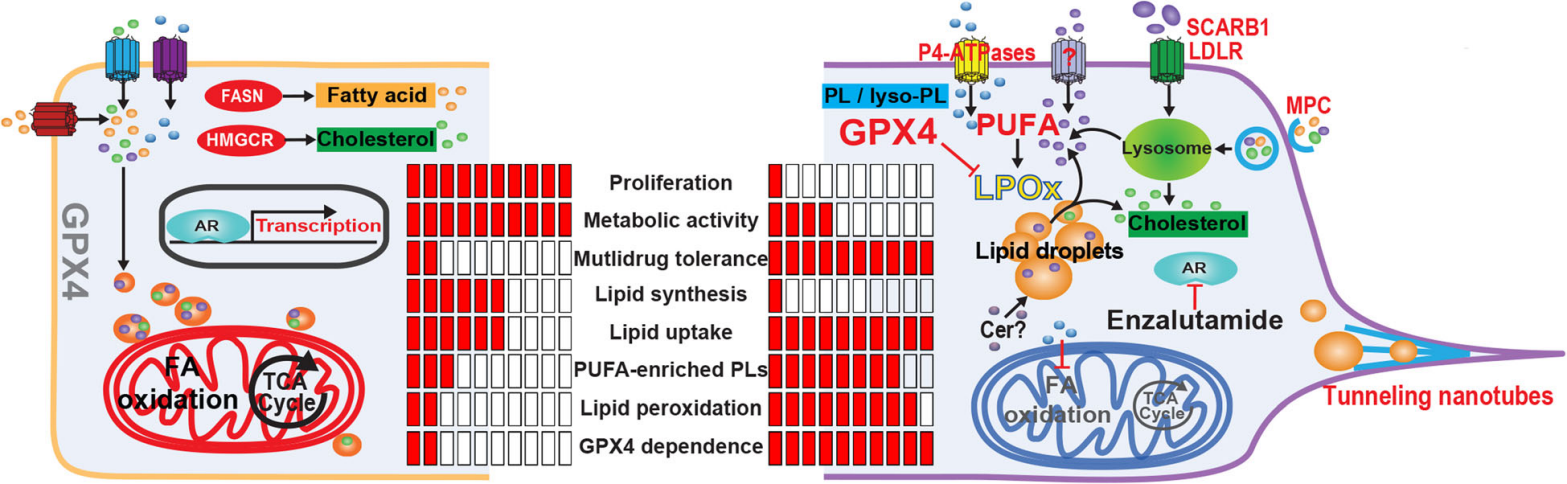
Graphic representation of changes to lipid supply and lipid remodeling that underpin therapy-induced multidrug tolerance and ferroptosis hypersensitivity in PCa cells. In the androgen-stimulated, proliferative state (left), the cellular lipid pool is fueled by both de novo lipogenesis (DNL) of cholesterol (green) and FAs (orange) and lipid uptake. Mitochondrial FA oxidation and TCA cycle activity provide energy and precursor for biomass synthesis. In the therapy-induced quiescent state, DNL is reduced, whereas lipid uptake through cargo-selective (lipid transporters) and non-selective transport mechanisms (tunneling nanotubes and macropinocytosis, MPC) is enhanced. Therapy-induced lipid remodeling includes mobilization of storage lipids (CEs and TAGs) through lipases, increased levels of all major phospholipid species and their enrichment with PUFAs (purple), leading to increased membrane fluidity, lipid peroxidation (LPOx), and GPX4 dependence. Increased and decreased activities are highlighted in red and gray, respectively. Cer, ceramides

Our longitudinal study of the early adaptive response to androgen deprivation therapy (ADT) and Enz (0–21 days) in PCa cells made several key discoveries related to reprogrammed lipid metabolism (summarized in Fig. 6). Moreover, our work provided several novel potential targets for intervention strategies to combat ATT resistance that needs pre-clinical evaluation. Importantly, our study is first in class to demonstrate that therapy-induced lipid remodeling and lipid supply plasticity are critical mechanisms that underpin GPX4 dependence and ferroptosis hypersensitivity in persister cells. Thus, our findings might have wider implications for our understanding of early events of therapy resistance since GPX4 dependence has been reported in several types of cancer in response to different targeted therapies [9, 49].

Consistent with previous studies that showed that subpopulations of breast and lung cancer cells, called drug-induced persister cells, entered a quiescent state in response to anti-cancer treatments ([9, 49] and reviewed in [55]), our functional studies showed that ATT-induced cellular quiescence was characterized by decreased proliferation, ATP production, and mitochondrial activity (Figs. 2c, S2C, and S2D). Previous studies have also reported a quiescence state of surviving cells after 3 weeks of androgen deprivation [6, 33, 34] and re-initiation of proliferation after ~ 6 months of treatment [6].

Unexpectedly, in light of cellular quiescence and low bioenergetic activity, Enz-induced remodeling of all major lipid classes (an energy-intensive process) was characterized by substantial accumulation of phospholipids and depletion of typical storage lipids (TAGs and CEs, Figs. 3 and S3) resident in lipid droplets (LDs). Yet, LD number and size (Fig. 3b) were increased. Therapy-induced LD formation in the context of growth inhibition has been previously demonstrated in cancer cells in response to oncogenic signaling inhibition [56]; however, the lipid composition of LDs was not delineated. It was recently shown that ceramides are converted to acylceramides via DGAT2 and stored in lipid droplets [57]. While DGAT1 and DGAT2 are both involved in TAG synthesis, DGAT2 also drives acylceramide synthesis and subsequent storage in lipid droplets. DGAT2 expression was upregulated in Enz-treated cells while DGAT1 was downregulated (Table S1), suggesting that increased acylceramide synthesis and lipid droplet storage caused the measured changes to lipid droplet morphometry. Ceramide metabolism was also highlighted by our comparative gene signature analysis (Fig. S1C). Given the heterogeneous nature of lipid droplets within cell populations [58] and that their accumulation has recently been described as a mechanism of drug resistance in colorectal cancer [59], renal cell carcinoma [56], and breast cancer cell lines [60], further investigations into lipid droplet biology under therapy stress are warranted.

Stable isotope carbon tracing revealed little glycolytic contribution to FA and cholesterol de novo synthesis (data not shown) and a 70% reduction of cholesterol synthesis from acetate-derived carbon (Fig. 4a). While lipogenesis can be fueled by other carbon sources (glutamine and FAs), enhanced lipid synthesis from alternative fuels was not supported by the measured bioenergetic, redox, and mitochondrial activity statuses (Fig. 2c). Moreover, the transcriptome and proteome changes of Enz-treated LNCaP cells collectively indicated a reduction in oxidative phosphorylation (Fig. 2b) and, more importantly, a decrease of multiple key enzymes of de novo FA and cholesterol biosynthesis (Fig. 4b). These results were consistent with reduced toxicity of several lipogenesis inhibitors (Figs. 2d and S2G). Instead, therapy-induced lipid remodeling and increased lipid content was fueled by enhanced lipid uptake via cargo-selective (transporters, Fig. 4c–f) and non-selective uptake mechanisms (macropinocytosis and TNTs, Figs. S4E and S4F). We frequently observed mitochondria and lipid droplets within Enz-induced TNTs (Fig. S4F), suggesting that these conduits of intercellular transport might contribute considerable amounts of lipid biomass. Similarly, macropinocytosis of cell debris including membrane components have been previously shown to significantly contribute to the cellular lipid pool [45]. Notably, the lipid supply switch to uptake excluded uptake of saturated FAs as indicated by reduced uptake of C16:0-Bodipy after extended ATT treatments (Enz and ADT, Fig. 4g), indicating that other uptake mechanisms or transporters were responsible for the increased cellular levels of saturated FAs and PUFAs. Together, these results demonstrate that ATTs induced a switch of lipid supply from a mix of de novo synthesis and exogenous sources to predominantly uptake of exogenous lipids via multiple mechanisms. Thus, lipid supply plasticity is a novel metabolic mechanism associated with ATT resistance development in PCa, and enhanced lipid scavenging can serve purposes other than biomass and energy production to support malignant cell proliferation. It is therefore critical to acknowledge that lipid supply plasticity during the development of ATT resistance limits the therapeutic window of individual treatments directed against either uptake or synthesis.

Only recently has attention been given to lipid uptake and to map the lipid transporter landscape in cancer cells [42, 61], including our own work in PCa [16]. The observed Enz-induced increase in protein expression of both LDLR and SCARB1 along with increased transcript levels of several other lipid transporters (Figs. 4e, f) supported the functional measurements obtained by qSCI of fluorescent lipid probes. They were concordant with previously reported transcriptional changes observed in our LNCaP tumor xenograft progression model in which recurring and CRPC tumors expressed higher levels of several lipid transporters and contained higher lipid content, including PUFAs, compared to tumors resected before castration [32]. Furthermore, increased expression of several lipid transporters were also observed in bone metastasis of CRPC patients (Fig. S4B, [16]); however, whether this is indirectly caused by the lipid-rich environment present in adult bone tissue (high adiposity) or directly by ATTs remains to be shown.

A major limitation of pharmacological targeting of PCa lipid supply is the scarcity of inhibitors and the incomplete understanding of mass contributions of different lipid supply pathways to the cellular lipid pool and their redundancies and cross-talk and compensatory capacities in response to reduced lipid synthesis, i.e., lipid supply plasticity. Lipogenesis on the other hand has remained a major target in cancer therapy, yielding an arsenal of inhibitors, unfortunately, with limited clinical success. There is increasing acknowledgement that various lipid scavenging pathways, including transporter-mediated uptake, tunneling nanotubes [46], lipid-conjugated albumin uptake [62], and macropinocytosis [18], are critical supply routes of exogenous lipids fueling bioenergetic processes and biomass production that underpin tumor growth and survival. Recent studies of cancer cells expressing the lipogenic phenotype, including PCa, estimated that > 70% of the lipid-derived carbon biomass is derived from uptake and only 30% from synthesis based on carbon tracing of serum-derived free FAs [63, 64]. However, these estimates are limited by the fact that the serum lipidome is highly complex and that > 95% of serum FAs are acyl conjugates across all lipid classes, including TAGs, CEs, and phospholipids. Thus, the carbon contribution from the collective uptake of all lipid classes to lipid biomass in proliferating cancer cells is likely to be even higher than the above estimates. However, due to above reasons, the delineation of the mass contribution of individual uptake mechanisms will be technically challenging.

Despite these current limitations, we showed that blocking cholesterol uptake via NPC1 inhibition with U18666A (Fig. S2H) is a promising strategy to exploit the increased demand of ATT-treated PCa cells for exogenous cholesterol (Fig. 4c). Cholesterol is a critical storage lipid (as acyl-ester in lipid droplets) and serves as a precursor for the steroidogenic pathway in which testosterone can be endogenously synthesized in PCa tumors [65] and that activation of the steroidogenic pathway is an ATT-induced adaptive response contributing to CRPC progression despite castrate levels of circulating androgens [66–68]. Additionally, non-esterified, membrane cholesterol is shown to affect lipid raft composition which may influence oncogenic signaling [69], further highlighting the critical dependence of PCa cells on cholesterol for growth and survival, particularly under therapy stress. However, targeting cholesterol supply as a therapeutic strategy in PCa solely focused on the inhibition of de novo cholesterol synthesis, i.e., inhibition of the early steps of the mevalonate pathway (HGMCR with statins) [70]. Thus, co-targeting of lysosomal processing of exogenous cholesterol with U18666A and synthesis pathways with statins in the context of ATTs may prove a potent strategy that targets aforementioned aspects of cholesterol metabolism as well as sensitizes to ferroptosis through loss of intermediates of the mevalonate synthesis pathway (i.e., isopentenyl pyrophosphate and farnesyl diphosphate) that are critical for GPX4 [11] and FSP1 activities [13, 14]. Oxidative stress induction by co-targeting the mevalonate pathway and the glutathione pool via cystine import inhibition has recently been proven successful in causing profound tumor cell death in pancreatic cancer [71].

In addition to Enz-enhanced lipid uptake and content, our work described extensive lipid remodeling that was characterized by increased FA elongation and desaturation (Fig. 5a) of phospholipids. Both processes have previously been associated with PCa incidence and aggressiveness [72, 73] and have been reported to promote survival in therapy-resistant ovarian cancer stem cells [74]. Phospholipids containing long-chain FAs along with membrane-bound cholesterol are critical for membrane stabilization and are involved in lipid raft formation where oncogenic growth signaling occurs [69, 73]. Enz-induced high PUFA levels in phospholipids were associated with increased membrane fluidity (Fig. 5b) which is thought to play a role in reduced sensitivity to chemotherapy reagents (i.e., multidrug tolerance, Fig. 2d) [75] and increases permeability and cell mobility and facilitates membrane-centered biological processes (vesicle formation, membrane fusion, cell-cell interaction, modulation of membrane enzymes, receptors, and transporters) [76]. Increased PUFA content of phospholipids does, however, generate a metabolic vulnerability [48]: increased oxidative damage through reactive oxygen and nitrogen species (ROS/RNS)-induced lipid peroxidation (Fig. 5d). Recent work showed that exogenous PUFAs can sensitize triple negative breast cancer to ferroptosis [77]. If not enzymatically repaired by GPX4 at the expense of reduced glutathione, accumulation of lipid hydroperoxides can lead to cell death via ferroptosis. Consistent with this, Enz-treated PCa cells showed reduced reductive power and hypersensitivity to selective GPX4 inhibitor, RSL3 (Fig. 5e). ADT and Enz-induced sensitivity to GPX4 inhibition of PCa cells was reminiscent of lapatinib- and vemurafenib-induced drug tolerance and emergence of “persister cells” in melanoma, breast, lung, and ovarian cancer [9] and therapy-resistance-associated high-mesenchymal state cancer cells [78]. It was speculated that GPX4 dependence in these therapy-induced states relied on the FA composition of the lipid bilayers. Our results of therapy-induced lipid remodeling causing PUFA enrichment provide first-in-class evidence to support this hypothesis. More importantly, the diversity of targeted therapies causing this shared GPX4 dependence suggests that lipid remodeling leading to PUFA enrichment of phospholipids might be a common mechanism associated with therapy resistance that could be pursued as therapeutic target in multiple types of cancer. However, the treatment window of therapy-induced ferroptosis hypersensitivity is likely to be restricted to the low cycling, quiescence phase [9] because high membrane PUFA levels sensitize to ferroptosis [77] and would limit proliferation rates [48] due to the increased lipid peroxidation stress generated by higher metabolic activities and associated ROS production. Such stress would be further aggravated by higher demands in reducing power (NADPH) for the recycling of oxidized glutathione and de novo lipid synthesis.

Therefore, impeding the acquisition of the therapy-induced persister cell phenotype by co-targeting one or several lipid remodeling processes might be a more promising strategy that might extend the efficacy of existing cancer therapies and delay resistance than combatting fully developed and often complex resistance mechanisms. By using enhanced GPX4 dependence (RSL3 sensitivity) as a biomarker of the persister cell phenotype, we demonstrated that co-targeting of several different processes of lipid remodeling protected ATT-treated PCa cells from ferroptosis hypersensitivity (Figs. 5g and S5G). In particular, the broad spectrum lipase inhibitors orlistat and CAY10499 [51] and FADS2 delta-6 desaturase inhibitor SC26196 [52] displayed high protective efficiencies against RSL3-induced cell death in ENZ-treated LNCaP cells when compared to Trolox. This demonstrated that therapy-induced GPX4 dependence can be modulated by rationally informed co-treatments targeting lipid remodeling. Whether suppression of therapy-induced GPX4 dependence alters the course of ATT resistance remains to be shown. We noted that mRNA expression of the majority of FA elongases and desaturases, including SCD1 and FADS2, were reduced after Enz treatment for 7–21 days (Fig. 5c). Yet, inhibitors against these enzymes efficiently suppressed Enz-induced ferroptosis hypersensitivity when co-treated for 7 days (Figs. 5g and S5G). Thus, the different treatment periods might explain the divergence between inhibitor activity and target expression, i.e., Enz induces a time-dependent switch from endogenous PUFA synthesis by FA elongation and desaturation to enhanced uptake of exogenous PUFAs as demonstrated for uptake of other lipid species (Figs. 4c, d). Notably, FADS2 activity has been recently implicated in cancer plasticity [74, 79] and FADS2 inhibition with SC26196 impaired cancer stemness, NFκB signaling, and tumor initiation capacity [74]. Our work indicates a potential functional link between FADS2 activity and therapy-induced GPX4 dependence that warrants further investigations. Whether co-targeting of lipid remodeling in combination with ATTs ultimately delays resistance development is a priority for further studies. Furthermore, the ferroptosis altering effects of PUFA and antioxidant supplementation might have implications for PCa patients undergoing ATT treatment, i.e., changing the duration until therapy failure, that need evaluation.

It is clear from this work that, despite its numerous discoveries, there are many unanswered questions regarding mechanisms of therapy-induced lipid remodeling (e.g., if changes to lipidome are reverted upon acquisition of resistance) and how this process can be harnessed to extend efficacies of existing, clinically proven therapies. The most prevalent are confirmation of the PUFA-enriched phospholipid phenotype in other types of cancer displaying therapy-induced GPX4 dependence [9, 78], investigating if dietary aspects like PUFA consumption and antioxidant supplementation affect resistance development and pre-clinical testing of co-therapies against lipid remodeling to combat ATT resistance development and disease progression to currently lethal CRPC.

## Conclusions

Breast, melanoma, lung, and ovarian cancer treated with different therapies develop GPX4 dependence and ferroptosis hypersensitivity, which are metabolic hallmarks of persister cells, an early, drug-induced state that is associated with acquired therapy resistance and tumor relapse. Our study in prostate cancer in response to androgen-targeted therapies demonstrated that GPX4 dependence and ferroptosis hypersensitivity of persister cells are associated with extensive lipid remodeling, including enhanced lipid uptake and PUFA enrichment of membrane lipids (Fig. 6). It showed that lipase and fatty acid desaturase activities are critical for persister cells to develop GPX4 dependence. Since enhanced GPX4 dependence is an adaptive state shared by several types of cancer in response to different therapies, our discoveries might have universal implications for our understanding of metabolic events that underpin acquired resistance and inform novel co-treatments against tumor relapse.

## Supplementary information

**Additional file 1.** Supplementary Info.

**Additional file 2.** Supplementary Figures.

**Additional file 3:** Supplementary Table S1.

**Additional file 4:** Supplementary Table S2.

**Additional file 5:** Supplementary Table S3.

**Additional file 6:** Supplementary Table S4.

**Additional file 7:** Supplementary Table S5.

**Additional file 8:** Supplementary Table S6.

## Data Availability

Normalized gene expression data of the microarray experiment have been deposited in NCBI’s Gene Expression Omnibus (GEO) and are accessible through GEO Series accession number GSE143408. The proteomics dataset, lipid mass spectrometry data, and CellProfiler analysis pipelines are available from the corresponding author on request.

## Abbreviations

ADT: Androgen deprivation therapy
AR: Androgen receptor
ATT: Androgen receptor-targeted therapy
CE: Cholesterol ester
Cer: Ceramide
CRPC: Castration-resistant prostate cancer
CSS: Charcoal-stripped serum
DNL: De novo lipogenesis
Enz: Enzalutamide
ESI-MS: Electrospray ionization mass spectrometry
FA: Fatty acid
FDR: False discovery rate
GC/MS: Gas chromatography tandem mass spectrometry
GSVA: Gene set variation analysis
INT: Intact
LC/MS: Liquid chromatography tandem mass spectrometry
LD: Lipid droplet
LDL: Low density lipoprotein
LPC: Lyso-phosphatidylcholine
LPOx: Lipid peroxidation
MDT: Multidrug tolerance
MFI: Mean fluorescence intensity
MPC: Macropinocytosis
NAD: Nadir
PC: Phosphatidylcholine
PCa: Prostate cancer
PE: Phosphatidylethanolamine
PG: Phosphatidylglycerol
PL: Phospholipid
PS: Phosphatidylserine
PUFA: Polyunsaturated fatty acid
qSCI: Quantitative single-cell imaging
RNS: Reactive nitrogen species
ROS: Reactive oxygen species
SM: Sphingomyelin
TAG: Triacylglycerol
TNT: Tunneling nanotube
