# Supplementary material for: Therapy-induced lipid uptake and remodeling underpin ferroptosis hypersensitivity in prostate cancer
Source: Cancer Metab. 2020 Jun 19;8:11. doi: 10.1186/s40170-020-00217-6 (PMC7304214; doi:10.1186/s40170-020-00217-6)
Supplement: Supplementary file 1 — Additional file 1. Supplementary Info. [file 40170_2020_217_MOESM1_ESM.docx]

Therapy-induced lipid uptake and remodeling underpin ferroptosis hypersensitivity in prostate cancer

Kaylyn D Tousignant^1#^, Anja Rockstroh^1^, Berwyck LJ Poad^3^, Ali Talebi^4^, Reuben SE Young^3^, Atefeh Taherian Fard^1^, Rajesh Gupta^3^, Tuo Zang^1^, Chenwei Wang^1^, Melanie L Lehman^1,5^, Johan V Swinnen^4^, Stephen J Blanksby^3^, Colleen C Nelson^1^ and Martin C Sadowski^1,2#*^

^1^ Australian Prostate Cancer Research Centre - Queensland, Institute of Health and Biomedical Innovation, School of Biomedical Sciences, Faculty of Health, Queensland University of Technology (QUT), Princess Alexandra Hospital, Translational Research Institute, Brisbane, Australia

^2^ Cancer & Ageing Research Program, Institute of Health and Biomedical Innovation, School of Biomedical Sciences, Faculty of Health, Queensland University of Technology (QUT), Translational Research Institute, Brisbane, Australia

^3^ Central Analytical Research Facility, Institute for Future Environments, Queensland University of Technology, Brisbane, Australia

^4^ Department of Oncology, Laboratory of Lipid Metabolism and Cancer, LKI Leuven Cancer Institute, KU Leuven-University of Leuven, Leuven, Belgium

^5^Vancouver Prostate Centre, Department of Urologic Sciences, University of British Columbia, Vancouver, Canada

#contributed equally

^*^corresponding author

**Supplementary Methods**

**Cell culture**

22RV1 (ATCC, CRL-2505) and DuCaP cells (ATCC, CVCL_2025) were cultured in RPMI medium (Thermo Fisher) supplemented with 5% and 10% FBS, respectively. Medium was changed every 3 days and cells were incubated at 37°C in 5% CO2. For treatments representing androgen-deprivation therapy, cells were cultured in RPMI supplemented with 5% charcoal stripped-serum (CSS). For AR targeted therapies, cells were cultured in 5% or 10% FBS with AR-antagonist Enzalutamide (Enz, 10 µM). Cells were passaged at approximately 80% confluency by trypsinization. Cell lines were genotyped in March 2018 by Genomics Research Centre (Brisbane) and routinely tested for mycoplasma infection.

**Measurements of cellular ATP levels and reductive power**

Cellular ATP levels and reducing power were measured by CellTiter-Glo luminescence and PrestoBlue fluorescence assays, respectively. Briefly, LNCaP cells cultured for the indicted times in either 5% FBS+Enzalutamide (10 µM) or 5% CSS were harvested by trypsinization and seeded into 96-well tissue culture plates (n=3 biological replicates with 4 wells/sample, Corning, Corning, NY, USA) at a density of 6,000 cells/well in their corresponding types of media (RPMI medium supplemented with either 5% FBS, 5%FBS+Enzalutamide (10 µM) or 5% CSS). After 48 hours, CellTiter-Glo and Presto Blue assays were performed according to the manufacturer’s instructions. Luminescence and fluorescence were in a FLUOstar Omega microplate reader (Bio Tek). Measurements were normalized based on total cell count by DNA staining with Hoechst 33342.

**Quantitative single cell imaging of metabolic parameters by fluorescent microscopy**

Prior to seeding, imaging plates were coated with 150 µl Poly-l-ornithine (PLO, Sigma) and washed with PBS to increase cell attachment. PCa cell lines pre-treated with either 5% FBS+Enzalutamide (10 µM) or 5% CSS were harvested three days prior completion of the indicated treatment times by trypsinization and seeded into PLO-coated 96-well Ibidi optical plates (n=3 biological replicates with 4 wells/sample) at a density of 6,000 cells/well in their corresponding types of media (RPMI medium supplemented with either 5% FBS (D0), 5%FBS+Enzalutamide (10 µM) or 5% CSS). To measure glucose uptake, media was removed after three days (completion of full treatment period), and cells were incubated at 37°C for one hour in 65 μL/well solution of glucose-free RPMI (Thermo Fisher) supplemented with 2-NBDG (2-(N-(7_Nitrobenz-2-oxa-1,3-diazol-4-yl)Amino)-2-Deoxyglucose, 50 μM, Thermo Fisher). After incubation, cells were washed and fixed with 4% PFA. Cellular DNA and F-actin was counterstained with DAPI and Alexa Fluor 647 Phalloidin (Thermo Fisher), respectively. Approximately 750 cells/well (3-8 wells/sample) were imaged. For measuring macropinocytosis, growth media was exchanged with 80 µL/well of serum-free RPMI media (0.2% lipid-free BSA) supplemented with 70 kDa dextran-TR (1 mg/mL, Thermo Fisher), and cells were incubated at 37°C for one hour. DNA and lysosomes were counterstained with Hoechst 33342 and LysoTracker Red DND-99 (5 µM, Thermo Fisher). After washing with serum-free media, cells were imaged live. Autophagy was measured by live imaging of ATT-treated cells stained with Cyto-ID reagent (2 μL/mL, Enzo Life Sciences) for 60 min at 37°C. Total mitochondrial mass per cell (independent of membrane potential) was measured by live imaging of MitoTracker Green FM-stained cells (200 nM, Thermo Fisher). All images were acquired with the InCell 2200 automated fluorescence microscope system (GE Healthcare Life Sciences) at 10x and 40x magnifications. Quantitative image analysis of mean fluorescence intensity (MFI) and morphometric data was performed with Cell Profiler Software (Broad Institute).

**GC-MS FAME analysis of serum**

Extractions of lipids from FBS and CSS (Sigma Aldrich) used for cell culture experiments in these studies were performed at a 9:1 MTBE:serum ratio with added internal standard (SPLASH Lipid-o-mix deuterated internal standard obtained from Avanti Polar Lipids, Alabaster, AL) and n-Nonadecanoic acid (C19:0) and briefly vortexed. 770 µL MTBE was added and mixture was incubated for 1 h at room temperature in a shaker. Phase separation was induced by adding 200 µL NH_4_CH_3_CO_2_ (150 mM). After vortexing for 20 s, the sample was centrifuged at 2,000 g for 5 min. The upper (organic) phase was collected and stored at -80°C, then diluted into 2:1 MeOH:CHCl_3_ with 7.5 mM NH_4_CH_3_CO_2_ for MS analysis.

Hydrolysis and derivatization of lipids extracts to fatty acid methyl esters (FAME) was performed on-line using trimethylsulfonium hydroxide [1]. FAMEs were analyzed with a gas chromatograph coupled to a mass spectrometer (GCMS – TQ8040; Shimadzu, Kyoto, Japan). The separation was carried out on a RTX-2330 capillary column (cyanopropyl stationary phase, 60 m x 0.25 mm, film thickness 0.20 μM; Restek, Bellefonte, PA, USA) and the electron ionization energy was set at 70 eV. Conditions for the analysis of FAMEs were as follows: carrier gas, He: column flow at 1 mL/min; 22:1 split ratio, injection volume 1 μL; injector temperature 240 ^o^C; interface and ion source temperature 260 ^o^C. GC oven temperature was maintained at 100 ^o^C for 1 min, thermal gradient 100 °C to 140 ^o^C at 10 ^o^C / min, 140 °C to 175 ^o^C at 6 ^o^C / min, 175 °C to 200 ^o^C at 10 ^o^C / min and hold for 1 min, followed by 200 °C to 250 ^o^C at 5 ^o^C / min and hold for 4 mins. The data were acquired with Q3 scan mode from *m/z* 50 – 650. For data collection the MS spectra were recorded from 4.6 min to 28.33 mins. The data was processed in GCMSsolution software (Shimadzy, Kyoto, Japan). Fatty acids were identified based on retention time alignment with reference FAMEs from a mixture of standards (Food Industry FAME mix (37 components), Restek, Bellefonte, PA USA). All samples were normalised to internal standard C19:0 and peak areas of relevant FAMEs was subtracted from negative control samples.

**Quantitative mass spectrometry of isobaric mass tag-labeled peptides**

LNCaP cells were treated with DMSO or Enzalutamide (10 µM) for 21 days as described above. Cell lysates from 3 independent experiments were prepared (6 samples) and peptides were labelled according to manufacturer’s protocol (ThermoFisher, cat. 90064). Briefly, proteins were precipitated overnight using 6 volumes of acetone. Dried pellet was resuspended in 50 mM TEAB, and samples were digested in trypsin overnight. Each sample was labeled with a different TMT label reagent (isobaric mass tag) for 1 hour, after which 5% hydroxylamine was added to quench the reaction and equal amounts of each sample were combined. Following C18 clean-up, 2 ug of total protein was injected for analysis on ESI QExactive HCD mass spectrometer. Data were analyzed using Proteome Discoverer^TM^ software (Thermo Fisher), and data integration of proteomics and transcriptomics data was carried out with MetaboAnalyst software (Mc Gill University).

**Statistical Analysis**

Statistical analysis were performed with Graphpad Prism 8.3 (Graphpad Software, San Diego, CA) and R Studio (RStudio, Boston, MA). Data reported and statistical tests are described in the figure legends.

**Supplementary Figure Legends**

**Figure S1**: **Longitudinal analysis of therapy-induced reprograming of metabolic networks in PCa xenograft model of CRPC progression and PCa patients pre- and post ADT**

(A) Gene set variation analysis (GSVA) of the transcriptome data obtained by microarray analysis of LNCaP tumor xenografts resected at the indicated therapy phases to monitor CRPC progression [2]: prior castration (intact, INT, n=10), regressing PSA post castration (REG, n=6), PSA nadir (NAD, n=10), rising PSA (recurring, REC, n=6), PSA exceeding intact (castrate-resistant, CR, n=6) [2]. The heatmap was generated with a hierarchical clustering algorithm using completed linkage and Euclidean distance measures and scaled by row z score (red=positive z score, blue=negative z score). The time points of tumor resection are indicated in the schematic. (B) Protein lysates of LNCaP cells treated for 0 and 21 days with Enz (10 µM) were trypsin digested, and peptides labelled with isobaric mass tag labels for quantitative proteomics analysis (n=3). Partial least squares (PLS) discriminant analysis shows the separate clustering of both treatments (top left panel). Volcano plot (top right panel) of differentially regulated proteins relative to day 0 (D0; FDR: fold change ≥2.0, p≤0.05). The 25 most significantly differentially expressed proteins are named (red label). Heatmap (bottom panel) showing fold-changes of the top 50 most significantly dysregulated proteins relative to D0 was generated with a hierarchical clustering algorithm using completed linkage and Euclidean distance measures and scaled by row z score (red=positive z score, blue=negative z score). (C) Indicated transcriptomics data sets from this study and study-relevant Gene Expression Omnibus data [3, 4] were analyzed by comparative signature scoring of lipid metabolism related gene sets using GSVA. Non-scaled bubble plots were created with the Morpheus webtool, with color indicating the direction of change in the GSVA score (red=increased scores/gene sets increase in overall expression, blue=decreased scores/gene sets decrease in overall expression; sample sources: gray=PCa patient, pink=LAPC9, yellow=LNCaP, orange=C4-2B; ENZ^R^=Enz resistant cell lines, CRPC=castrate resistant tumor xenograft after castration, 1^st^ and 2^nd^=series of transplantation, Post=patient samples 22 weeks after androgen deprivation therapy (ADT), Pre=matching patient samples prior ADT). Genes of the Li et al 2018 signatures [4] are listed in Table S4.

**Figure S2: Functional and morphological characterization of therapy-induced changes**

LNCaP cells were treated for the indicated times with Enz (10 µM), and (A) AR mRNA expression was analyzed by qRT-PCR (left panel, n=3). (B) Maintained suppression of AR signaling is exemplified by the heatmap of microarray data showing classical androgen regulated genes that were differentially expressed at indicated time points of Enz treatment. For hierarchical clustering, an algorithm of completed linkage and Euclidean distance measures was used, and heatmap was scaled by row z score (red=positive z score, blue=negative z score). (C) Metabolic activity of LNCaP cells treated for the indicated times with Enz (10 µM) or androgen-depleted media (CSS) was analyzed as a measure of cellular ATP levels by CellTiter-Glo assay (top left panel, n=3, mean±SD), reductive power by PrestoBlue (top right panel, n=3, mean±SD) and mitochondrial membrane potential (MMP) by qSCI of MitoTracker Orange CMTMRos (bottom left panel, n>9000 cells, mean±SD, results are a representative of three independent experiments). Representative images are shown (bottom right panel, 40x magnification). (D) Proliferation of cells treated as in B was assessed based on cell confluence measured by phase contrast imaging every 2 hours for 96 hours (IncuCyte, n=3, mean±SD). Representative images (10x magnification) taken at 96 h of IncuCyte experiment demonstrate morphological difference between control (D0) and 21 days of treatment with Enzalutamide (Enz D21). (E) The percentage of cell death (left panel) was calculated based on the ratio of dead cells (propidium iodide-positive) relative to total cell count (Hoechst 33342-positive) by qSCI (n>9000 cells, mean±SD, results are a representative of three independent experiments). Autophagy (right panel) was assessed in LNCaP cells treated for 14 days with Enz (10 µM) by qSCI of CytoID-stained cells (right, n>9000 cells, mean±SD, results are a representative of three independent experiments). (F) Morphometric analysis based on qSCI of F-actin staining (cell mask) to measure cell area (left), cell perimeter (middle) and major axis length through cell body (right) (n>9000 cells, results are a representative of three independent experiments). (G) C4-2B (top panel) and DuCaP cells (middle panel) were cultured in growth media without (FBS) or with Enz (10 µM, FBS+ENZ D14) for 14 days including the final 24 h in the presence of vehicle control (DMSO) or the indicated compounds (paclitaxel 2.5 µM, docetaxel 2.5 µM, doxorubicin 2.5 µM, etoposide 5 µM, edelfosine 7.5 µM, TOFA 60 µM, RSL3 1.5 µM). LNCaP cells (bottom panel) were cultured in androgen-depleted growth media (CSS)for 14 days and co-treated as above. The percentage of dead cells was calculated based on qSCI of Hoechst 3342 (total cell count) and propidium iodide (dead cells) staining (n=3 wells/treatment with >4000 cells/well, representative result of 2 independent experiments). (H) LNCaP (left panel) and C4-2B cells (right panel) were cultured in growth media (FBS), androgen-depleted growth media (CSS for 5 and 14 days) or growth media supplemented with Enz (10 µM, FBS+ENZ) for 10 and 14 days including the final 24 h in the presence of vehicle control (DMSO) or U18666A (50 µM). The percentage of dead cells was calculated based on qSCI as described in (G; n=3 wells/treatment of LNCaP cells and n=2 wells/treatment for C4-2B cells with >4000 cells/well, representative of 2 independent experiments). Protein expression analysis of NPC1 and NPC2 in PCa patient samples (bottom panel) of normal gland (gray), localized primary tumor and (blue) and bone metastasis (red) as reported in the Iglesias-Gato proteome data set [5]. Statistical analysis for A-H: mean±SD, ns=not significant, *p<0.05, **p<0.01, ***p<0.001, ****p<0.0001, One-way ANOVA followed by Dunnett’s multiple comparisons test compared to D0/FBS).

**Figure S3**: **Lipidomics demonstrate therapy-induced lipid remodeling of all major lipid species**

LNCaP cells were treated for the indicated times with Enz (10 µM), and lipids were extracted and analyzed by LC/MS shotgun lipidomics (n=2 with 3 technical replicates each=6 data points). (A) Principal component analysis (PCA) with MetaboAnalyst software [6] showed clustering of the four time points of Enz treatment (FBS=D0 of Enz treatment, Enz D7, Enz D14 and Enz D21). LC/MS analysis of fold-changes of (B) sphingomyelines (SM), (C) phosphatidylcholines (PC), (D) phosphatidylethanolamine (PE), (E) phosphatidylserines (PS), (F) phosphatidylglycerols (PG), (G) cholesterol-esters (CE) and (H) triacylglycerols (TAGs) in indicated samples relative to FBS/D0 (n=2, mean±SD, Two-way ANOVA followed by Tukey’s multiple comparisons test). (I) Heatmap of the top 50 dysregulated lipid species relative to D0 (identified by One-way ANOVA and Fisher’s least significant difference method) was generated with a hierarchical clustering algorithm using completed linkage and Euclidean distance measures and scaled by row z score (red=positive z score, blue=negative z score; LPC=lyso-phosphatidylcholine).

**Figure S4**: **ATTs reduce glucose uptake but increase lipid transporter expression and cargo-nonselective mechanisms of nutrient uptake**

(A) Following 14 days of Enz treatment (10 µM), glucose uptake of LNCaP and C42B cells was measured by qSCI of NBD-2DG (n>9000 cells, mean±SD, results are a representative of 3 independent experiments). (B) Protein expression analysis of indicated lipid transporters and two lipogenesis enzymes (FASN and HMGCR) in PCa patient samples of normal gland (gray), localized primary tumor and (blue) and bone metastasis (red) as reported in the Iglesias-Gato proteome data set [5]. (C) LNCaP cells were treated for the indicated times with Enz (10 µM), and fatty acid content was analyzed by GC/MS FAME (n=2, mean±SD). (D) Fatty acid composition of fetal bovine serum (FBS) and charcoal-stripped FBS (CSS) used in these studies was analyzed by GC/MS FAME. Cholesterol and TAG concentrations are according to the supplier’s certified analysis. (E) LNCaP cells were treated as described in (A), and macropinocytosis was measured by qSCI of 70 kDa dextran-TR (n>9000 cells, results are a representative of 2 independent experiments). (F) LNCaP cells were treated for the indicated times with Enz (10 µM), and the presence of tunneling nanotubes (TNTs) connecting cells was measured by counting 100 F-actin stained cells (top left panel). Representative images of Enz D21 stained with MitoTracker Orange CMTMRos (yellow) and Bodipy (green) are shown (40x magnification). Arrows highlight TNTs containing active mitochondria and lipid droplets. Fold-change of mRNA expression of genes regulating TNT maturation and maintenance based on microarray analysis are shown (bottom panel, n=3). Statistical analysis for A, D and E: mean±SD, *p<0.05, **p<0.01, ***p<0.001, ****p<0.0001, One-way ANOVA followed by Dunnett’s multiple comparisons test compared to D0).

**Figure S5: Integrated fatty acid lipidomics and transcriptomics analysis and longitudinal analysis of LNCaP tumor xenograft model of CRPC progression highlight ATT-induced reprograming of PUFA metabolism**

(A) Integrated analyses (top panel) of dysregulated fatty acid species detected by GC/MS FAME (Fig. S4C) or (bottom panel) proteins detected by protein mass spectrometry (Fig. S1B) with transcriptomics data (Fig. 2) of LNCaP cells treated with vehicle control (FBS) or Enz (10 µM) for 21 days by MetaboAnalyst software 4.0 [6] (Mc Gill University) identified significantly enriched metabolic pathways of the Kyoto Encyclopedia of Genes and Genomes (KEGG) associated with PUFA metabolism. mRNA expression of indicated (B) fatty acid elongases and (C) fatty acid desaturases was measured by microarray in LNCaP tumor xenografts resected at the indicated therapy phases to monitor CRPC progression after castration [2] (see Figure S1 for more details) and were calculated relative to expression in tumors from sham-castrated mice (intact, n=10): regressing PSA post castration (REG, n=6), PSA nadir (NAD, n=10), rising PSA (recurring, REC, n=6), PSA exceeding intact (castrate-resistant, CRPC, n=6). The heatmaps showing all differentially expressed probes of the genes detected by microarray were generated with a hierarchical clustering algorithm using completed linkage and Euclidean distance measures and scaled by row z score (red=positive z score, blue=negative z score). (D) C4-2B (top left panel), DuCaP (top right panel), 22Rv1 (middle panel) were cultured in growth media without (FBS) or with Enz (10 µM, FBS+ENZ D14) for 14 days including the final 24 h in the presence of DMSO (0) or the indicated concentrations (0.5-1.5 µM) of GPX4 inhibitor RSL3. Lipid peroxide scavenger Trolox (100 µM) and ferroptosis inhibitor Ferrostatin-1 (10 µM) were used as controls to demonstrate cell death through ferroptosis. LNCaP cells (bottom panel) were cultured in androgen-depleted growth media (CSS) for 14 days and co-treated as above. The percentage of dead cells was calculated based on qSCI of Hoechst 3342 (total cell count) and propidium iodide (dead cells) staining (n=3 wells/treatment with >4000 cells/well, representative result of 2 independent experiments). (E) LNCaP (left panel) were cultured in FBS or androgen-depleted growth media (CSS) and C4-2B cells (right panel) were cultured or in FBS or FBS+ENZ (10 µM) for the indicated times (D=days) including the final 24 h in the presence of DMSO (0) or RSL3 (1.25 µM). The percentage of dead cells was calculated as described in D (n=3 wells/treatment with >4000 cells/well, representative result of 2 independent experiments). (F) Gene ontology analysis (Panther, reactome version 65) of 334 significantly changed proteins of the proteomics data set (Fig. S1B) show significant enrichment of pathways related to oxidative stress and selenocysteine metabolism. The complete full list is shown in Table S3. (G) LNCaP cells seeded in a 96 well plate were co-treated with the indicated compounds (orlistat 10 µM, CAY10499 17.5 µM, SC26196 30 µM, TOFA 2.5 µM, fatostatin 5 µM, arachidonic acid (AA) 20 µM, trolox 100 µM) and cultured in FBS or androgen-depleted growth media (CSS). After 7 days of co-treatment, cells were treated with vehicle control (DMSO/FBS) or RSL3 (1.25 µM) to assess GPX4 dependence by measuring cell death by qSCI as described in D (n=3 wells/treatment with >4000 cells/well, representative result of 2 independent experiments).

**References**

1. Butte, W., *Rapid method for the determination of fatty acid profiles from fats and oils using trimethylsulphonium hydroxide for transesterification.* Journal of Chromatography A, 1983. **261**: p. 142-145.

2. Locke, J.A., et al., *Arachidonic acid activation of intratumoral steroid synthesis during prostate cancer progression to castration resistance.* Prostate, 2010. **70**(3): p. 239-51.

3. Rajan, P., et al., *Next-generation Sequencing of Advanced Prostate Cancer Treated with Androgen-deprivation Therapy.* European Urology, 2014. **66**(1): p. 32-39.

4. Li, Q., et al., *Linking prostate cancer cell AR heterogeneity to distinct castration and enzalutamide responses.* Nature Communications, 2018. **9**(1): p. 3600.

5. Iglesias-Gato, D., et al., *The proteome of prostate cancer bone metastasis reveals heterogeneity with prognostic implications.* Clinical Cancer Research, 2018.

6. Chong, J., et al., *MetaboAnalyst 4.0: towards more transparent and integrative metabolomics analysis.* Nucleic Acids Research, 2018. **46**(W1): p. W486-W494.
