# Supplementary material for: Therapy-induced lipid uptake and remodeling underpin ferroptosis hypersensitivity in prostate cancer
Source: Cancer Metab. 2020 Jun 19;8:11. doi: 10.1186/s40170-020-00217-6 (PMC7304214; doi:10.1186/s40170-020-00217-6)

Fig S1A

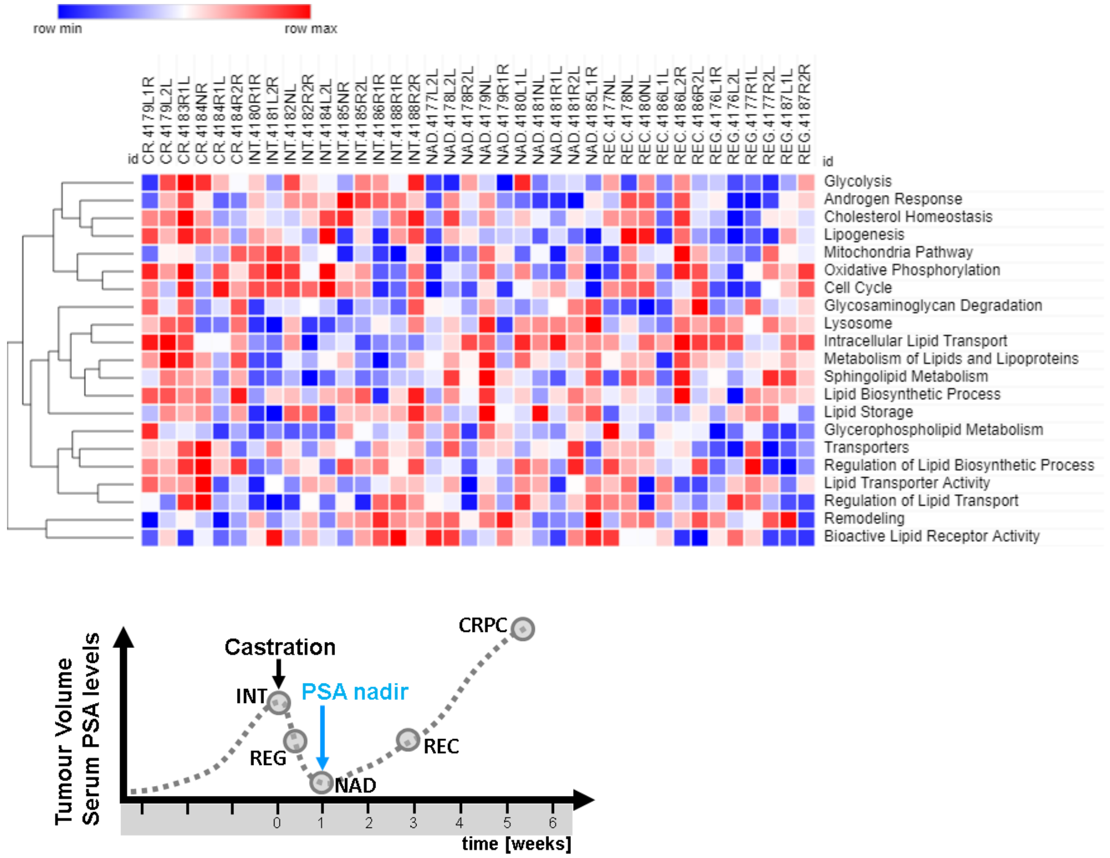
row min
row max
id
CR.4179L1R
CR.4179L2L
CR.4183R1L
CR.4184NR
CR.4184R1L
CR.4184R2R
INT.4180R1R
INT.4181L2R
INT.4182NL
INT.4182R2R
INT.4184L2L
INT.4185NR
INT.4185R2L
INT.4186R1R
INT.4188R1R
INT.4188R2R
NAD.4177L2L
NAD.4178L2L
NAD.4178R2L
NAD.4179NL
NAD.4179R1R
NAD.4180L1L
NAD.4181NL
NAD.4181R1L
NAD.4181R2L
NAD.4185L1R
REC.4177NL
REC.4178NL
REC.4180NL
REC.4186L1L
REC.4186L2R
REC.4186R2L
REG.4176L1R
REG.4176L2L
REG.4177R1L
REG.4177R2L
REG.4187L1L
REG.4187R2R
id
Glycolysis
Androgen Response
Cholesterol Homeostasis
Lipogenesis
Mitochondria Pathway
Oxidative Phosphorylation
Cell Cycle
Glycosaminoglycan Degradation
Lysosome
Intracellular Lipid Transport
Metabolism of Lipids and Lipoproteins
Sphingolipid Metabolism
Lipid Biosynthetic Process
Lipid Storage
Glycerophospholipid Metabolism
Transporters
Regulation of Lipid Biosynthetic Process
Lipid Transporter Activity
Regulation of Lipid Transport
Remodeling
Bioactive Lipid Receptor Activity
Tumour Volume
Serum PSA levels
Castration
CRPC
INT
PSA nadir
REG
REC
NAD
0
1
2
3
4
5
6
time [weeks]

Fig S1B

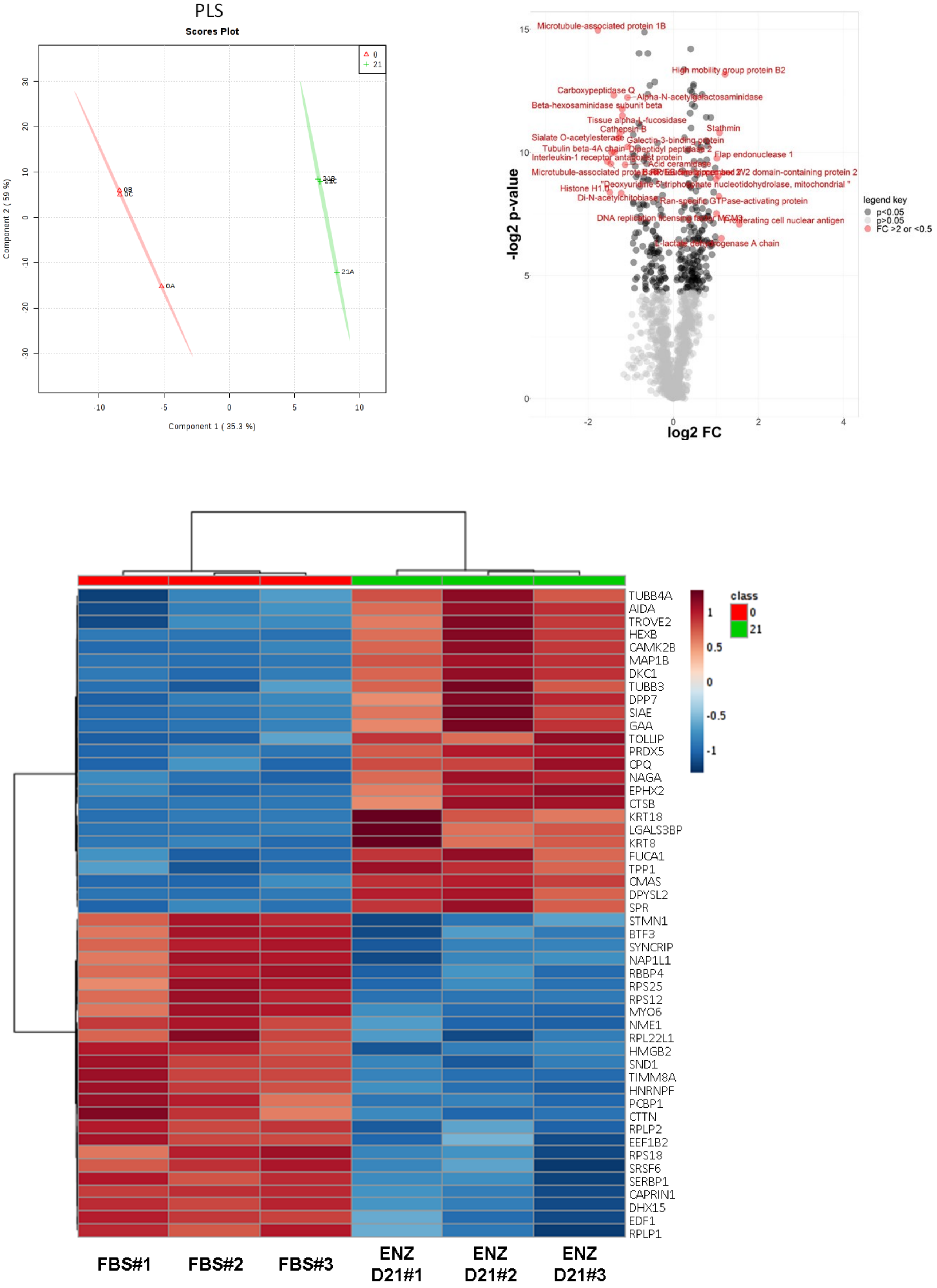

PLS
Scores Plot
0
21
0B
0C
0A
21B
21C
21A
Component 1 (35.3 %)
Component 2 (59 %)
-10
-5
0
5
10
30
20
10
0
-10
-20
-30
Microtubule-associated protein 1B
High mobility group protein B2
Carboxypeptidase Q
Alpha-N-acetylgalactosaminidase
Beta-hexosaminidase subunit beta
Tissue alpha-L-fucosidase
Cathepsin B
Stathmin
Sialate O-acetylesterase
Galectin-3-binding protein
Tubulin beta-4A chain
Dipeptidyl peptidase 2
Interleukin-1 receptor antagonist protein
Flap endonuclease 1
Acid ceramidase
Microtubule-associated protein
Histone H1.0
Di-N-acetylchitobiase
Ran-specific GTPase-activating protein
DNA replication licensing factor MCM3
Proliferating cell nuclear antigen
L-lactate dehydrogenase A chain
15
10
5
0
-2
0
2
4
-log2 p-value
log2 FC
legend key
p<0.05
p>0.05
FC >2 or <0.5
TUBB4A
AIDA
TROVE2
HEXB
CAMK2B
MAP1B
DKC1
TUBB3
DPP7
SIAE
GAA
TOLLIP
PRDX5
CPQ
NAGA
EPHX2
CTSB
KRT18
LGALS3BP
KRT8
FUCA1
TPP1
CMAS
DPYSL2
SPR
STMN1
BTF3
SYNCRIP
NAP1L1
RBBP4
RPS25
RPS12
MYO6
NME1
RPL22L1
HMGB2
SND1
TIMM8A
HNRNPF
PCBP1
CTTN
RPLP2
EEF1B2
RPS18
SRSF6
SERBP1
CAPRIN1
DHX15
EDF1
RPLP1
1
0.5
0
-0.5
-1
class
0
21
FBS#1
FBS#2
FBS#3
ENZ D21#1
ENZ D21#2
ENZ D21#3

Fig S1C

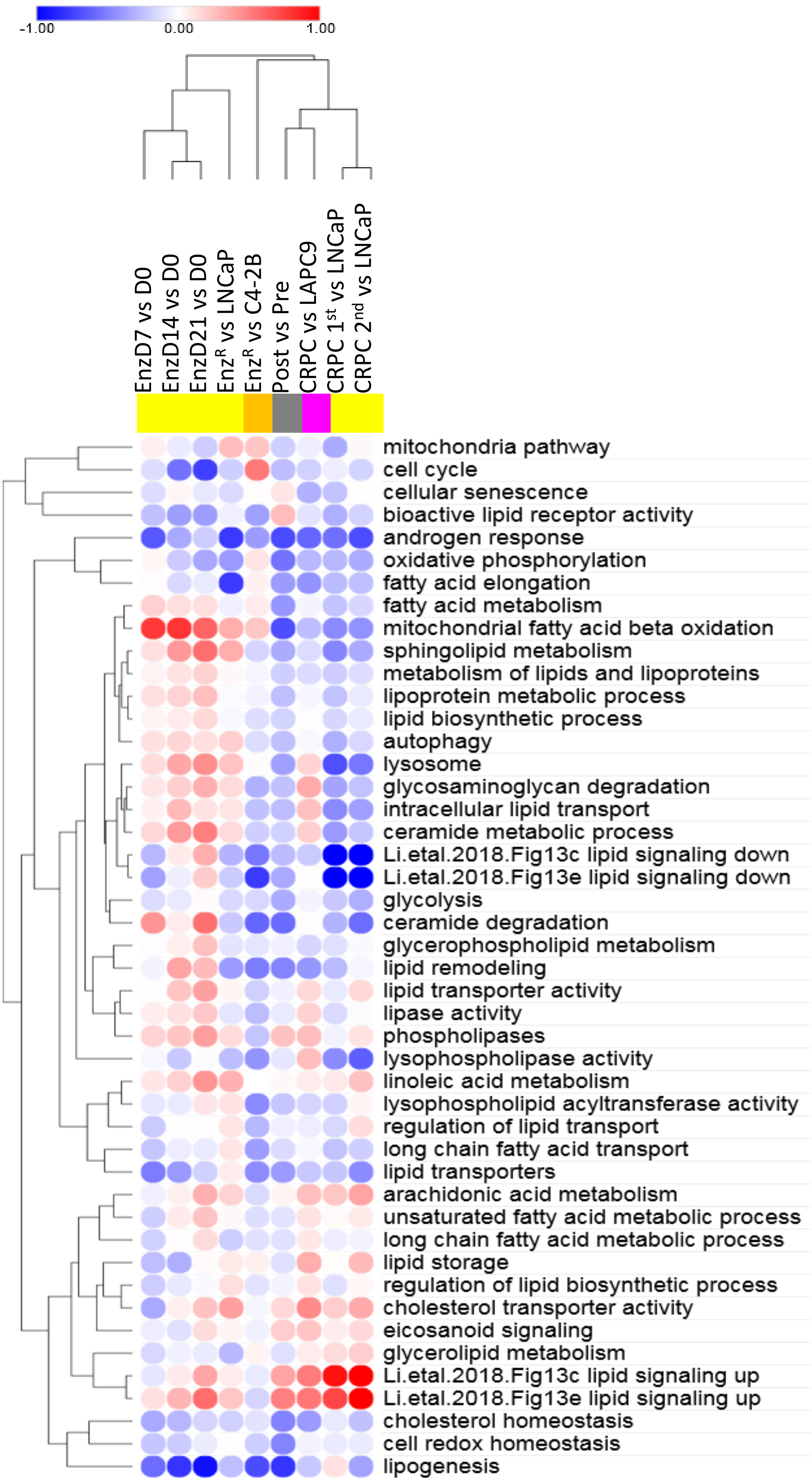

-1.00
0.00
1.00
EnzD7 vs D0
EnzD14 vs D0
EnzD21 vs D0
Enz^{R} vs LNCaP
Enz^{R} vs C4-2B
Post vs Pre
CRPC vs LAPC9
CRPC 1st vs LNCaP
CRPC 2nd vs LNCaP
mitochondria pathway
cell cycle
cellular senescence
bioactive lipid receptor activity
androgen response
oxidative phosphorylation
fatty acid elongation
fatty acid metabolism
mitochondrial fatty acid beta oxidation
sphingolipid metabolism
metabolism of lipids and lipoproteins
lipoprotein metabolic process
lipid biosynthetic process
autophagy
lysosome
glycosaminoglycan degradation
intracellular lipid transport
ceramide metabolic process
Li.etal.2018.Fig13c lipid signaling down
Li.etal.2018.Fig13e lipid signaling down
glycolysis
ceramide degradation
glycerophospholipid metabolism
lipid remodeling
lipid transporter activity
lipase activity
phospholipases
lysophospholipase activity
linoleic acid metabolism
lysophospholipid acyltransferase activity
regulation of lipid transport
long chain fatty acid transport
lipid transporters
arachidonic acid metabolism
unsaturated fatty acid metabolic process
long chain fatty acid metabolic process
lipid storage
regulation of lipid biosynthetic process
cholesterol transporter activity
eicosanoid signaling
glycerolipid metabolism
Li.etal.2018.Fig13c lipid signaling up
Li.etal.2018.Fig13e lipid signaling up
cholesterol homeostasis
cell redox homeostasis
lipogenesis

Fig S2A

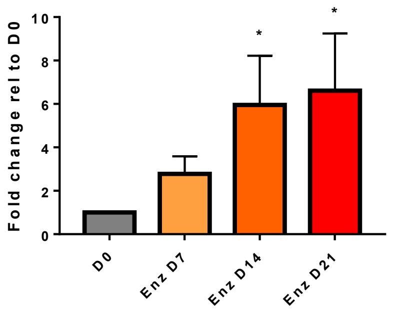

Fig S2B

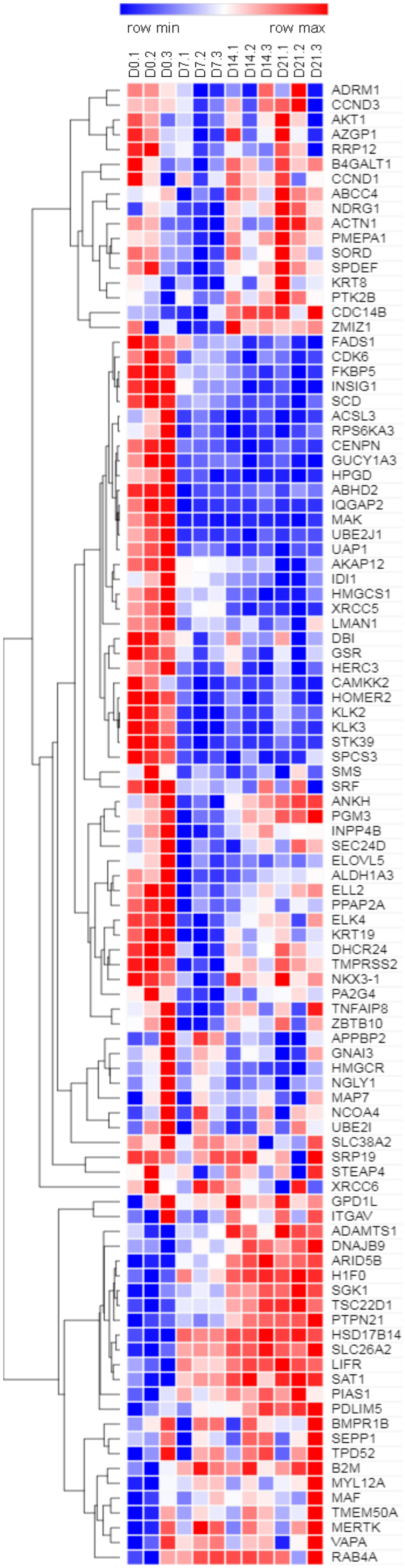

Fig S2C

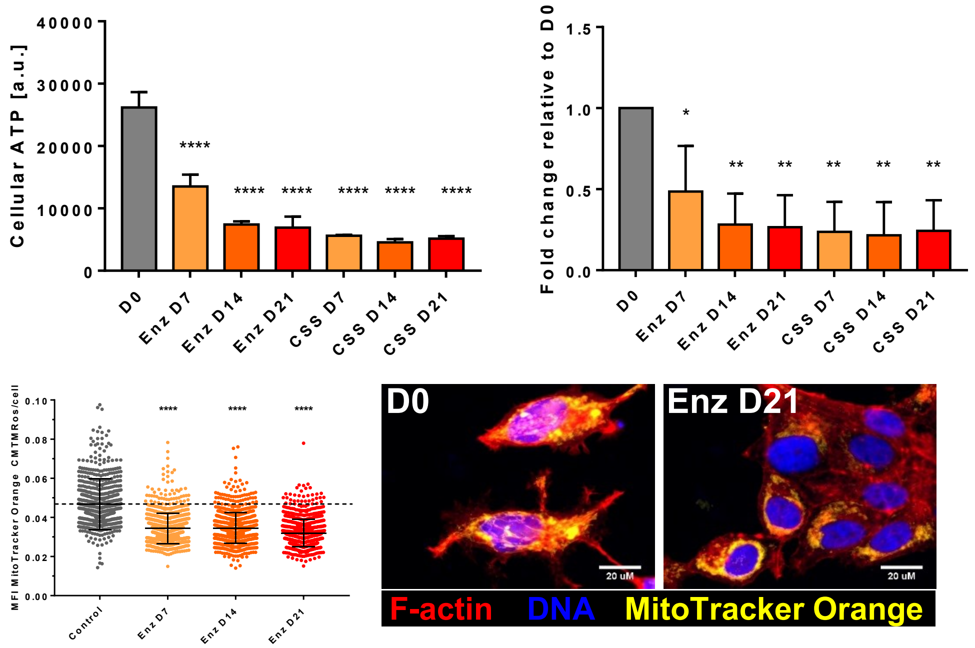

Fig S2D

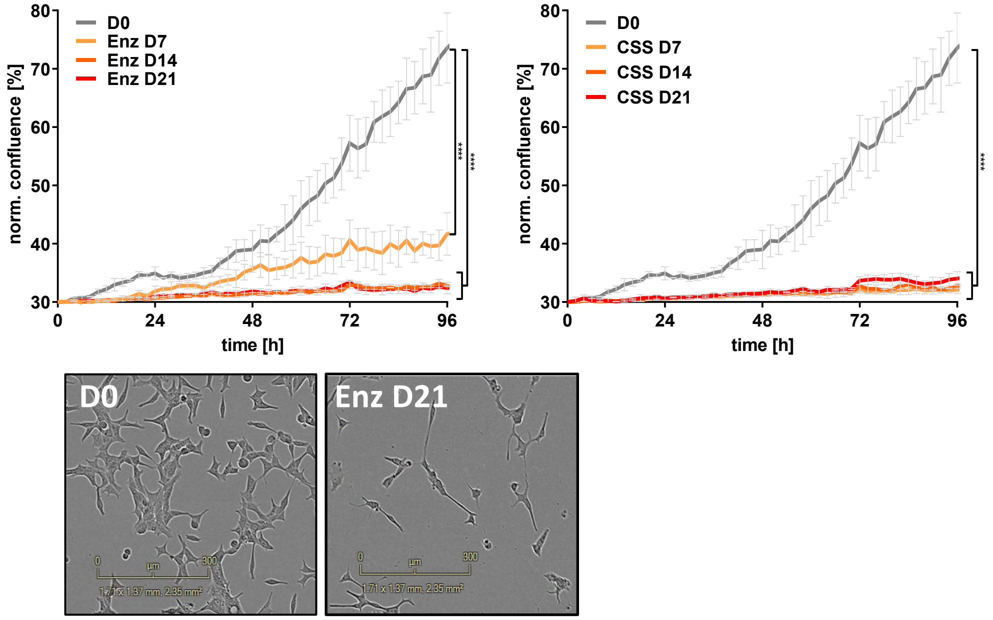

Fig S2E

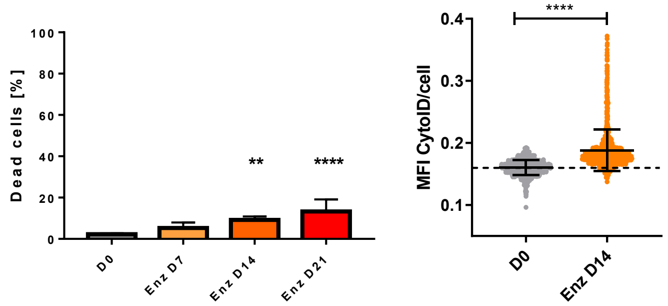

Fig S2F

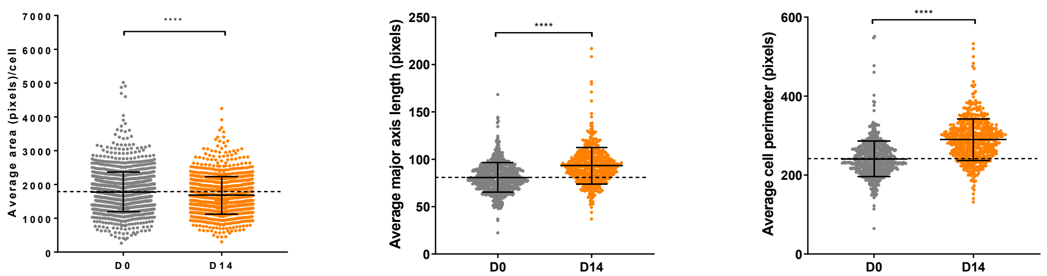

Fig S2G

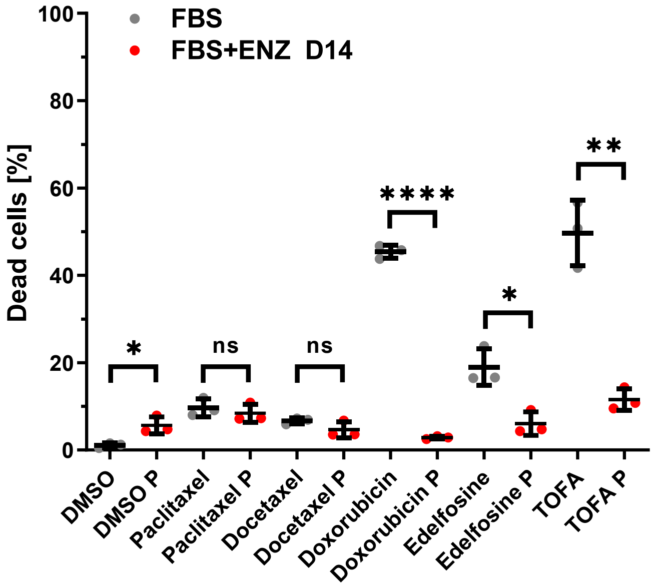
FBS
FBS+ENZ D14
Dead cells [%]
100
80
60
40
20
0
*
ns
ns
****
*
**
DMSO
DMSO P
Paclitaxel
Paclitaxel P
Docetaxel
Docetaxel P
Doxorubicin
Doxorubicin P
Edelfosine
Edelfosine P
TOFA
TOFA P

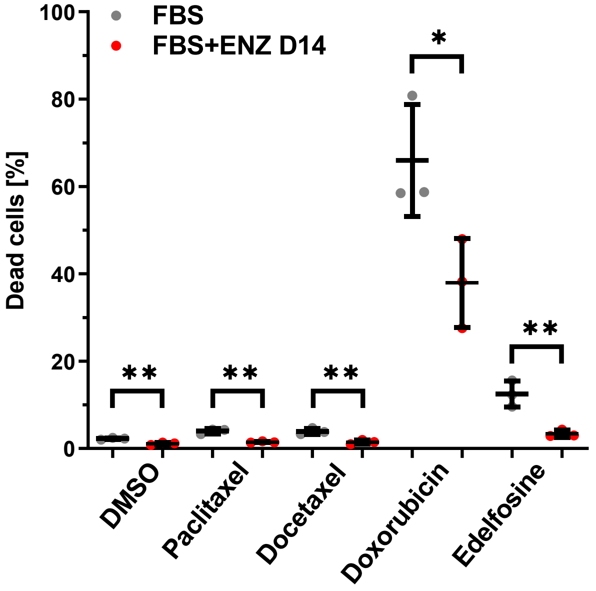
FBS
FBS+ENZ D14
Dead cells [%]
100
80
60
40
20
0
**
**
**
*
**
DMSO
Paclitaxel
Docetaxel
Doxorubicin
Edelfosine

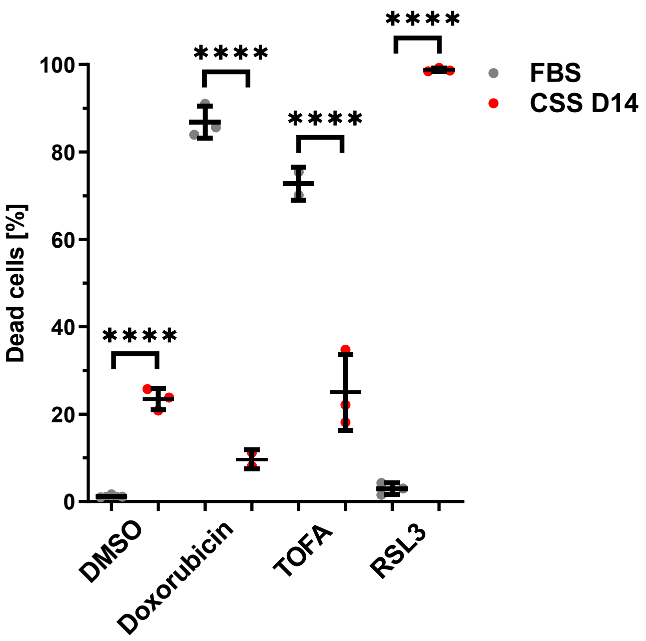
FBS
CSS D14
Dead cells [%]
100
80
60
40
20
0
****
****
****
****
DMSO
Doxorubicin
TOFA
RSL3

Fig S2H

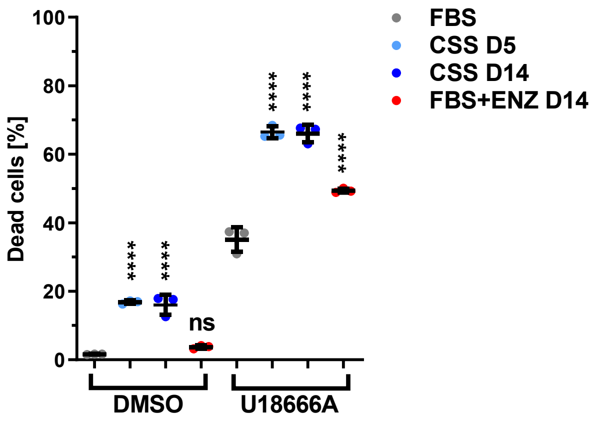
Dead cells [%]
100
80
60
40
20
0
****
****
ns
****
****
****
DMSO
U18666A
FBS
CSS D5
CSS D14
FBS+ENZ D14

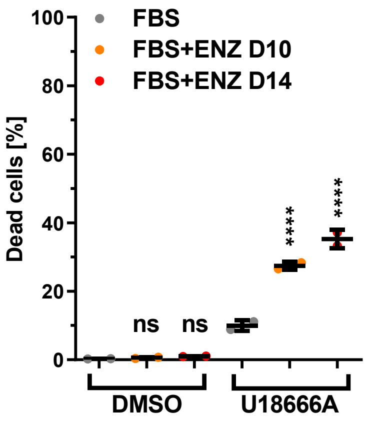
Dead cells [%]
100
80
60
40
20
0
ns
ns
****
****
DMSO
U18666A
FBS
FBS+ENZ D10
FBS+ENZ D14

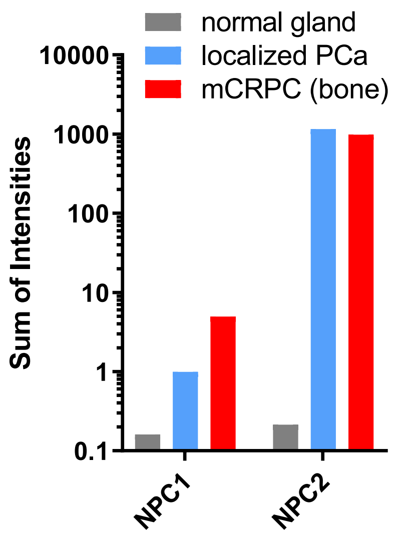
normal gland
localized PCa
mCRPC (bone)
Sum of Intensities
10000
1000
100
10
1
0.1
NPC1
NPC2

Fig S3A

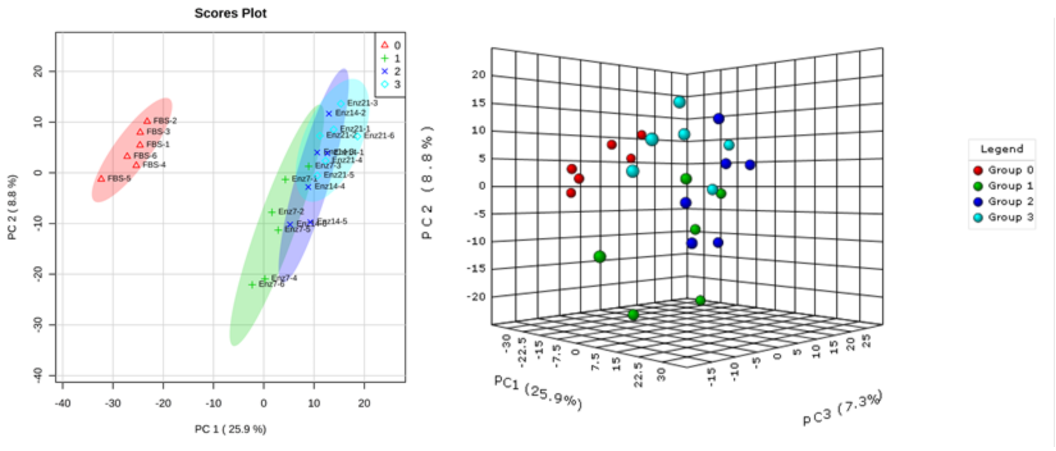

Fig S3B

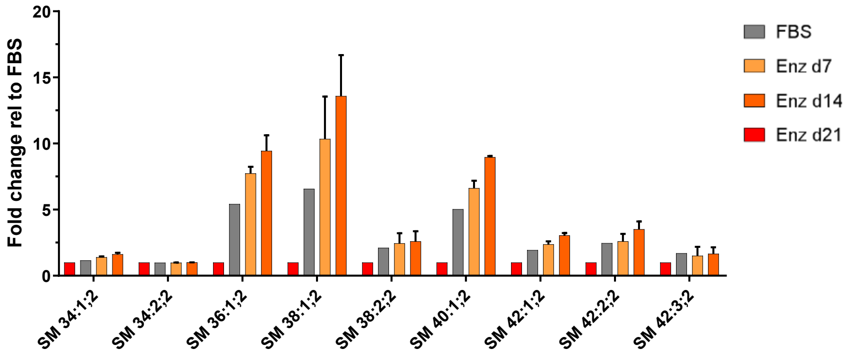

Fig S3C

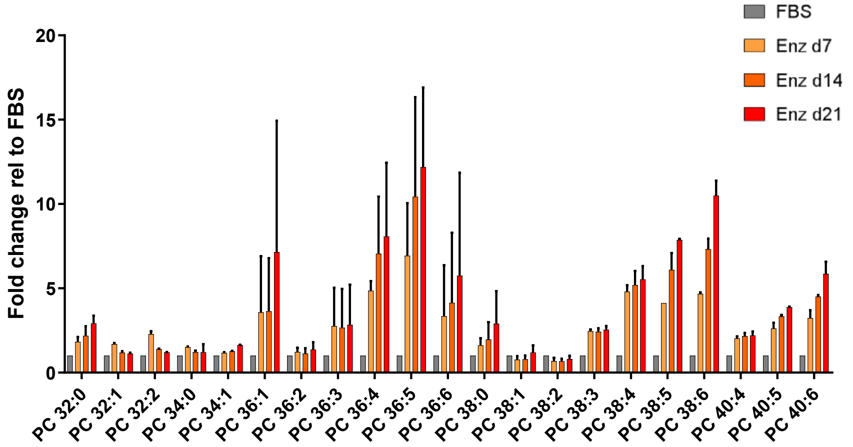

Fig S4D

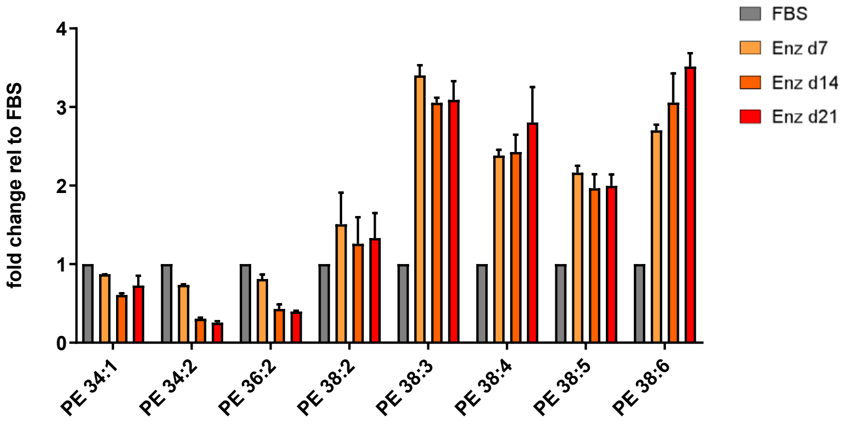

Fig S3E

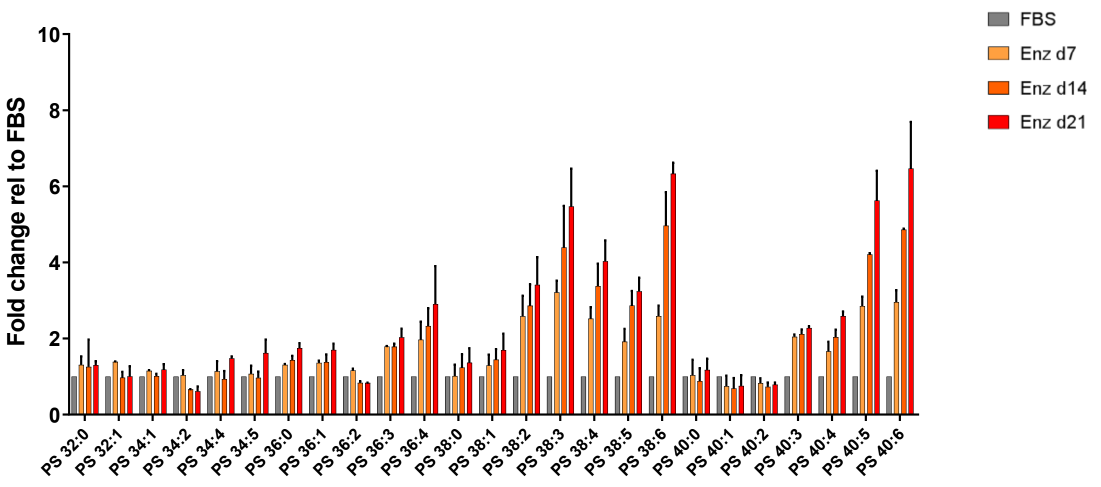

Fig S3F

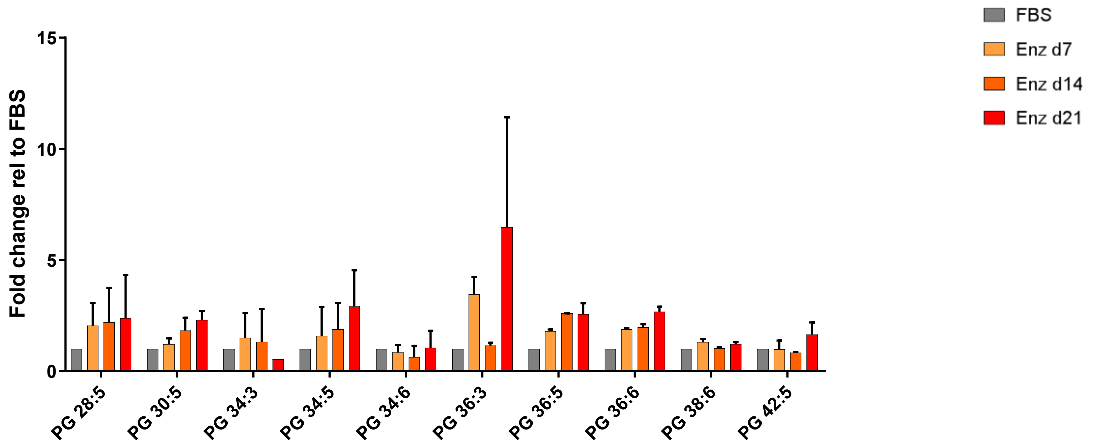

Fig S3G

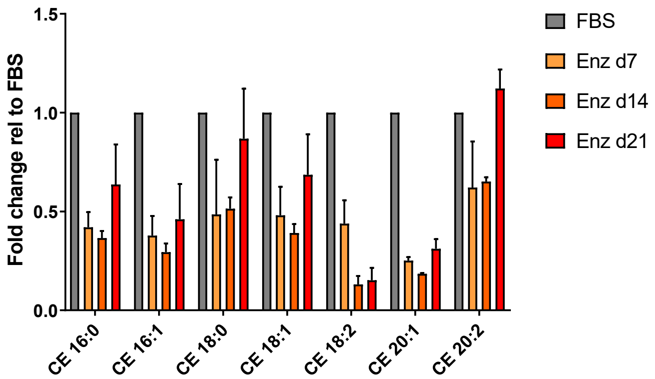

Fig S3H

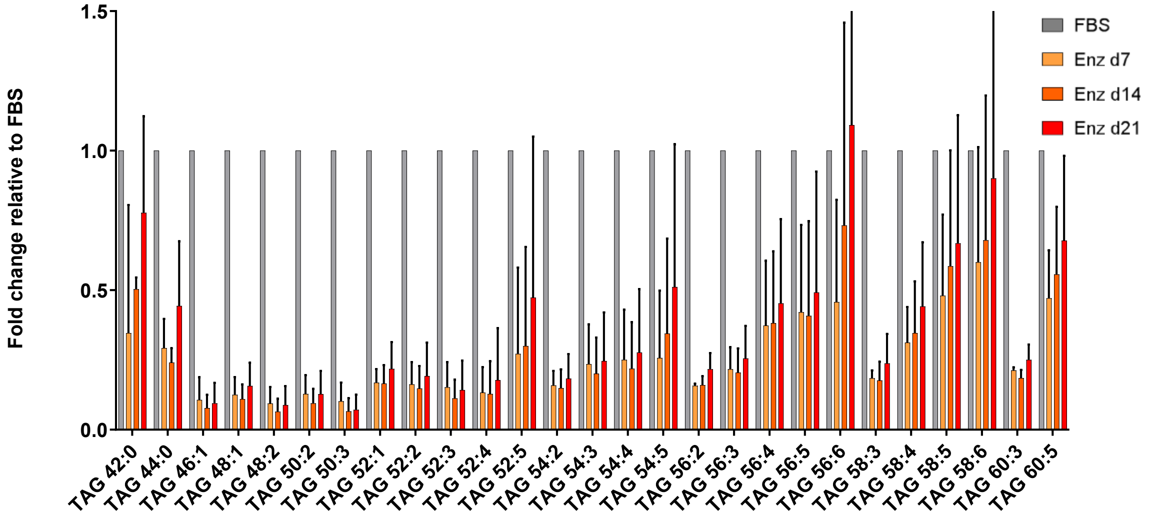

Fig S3I

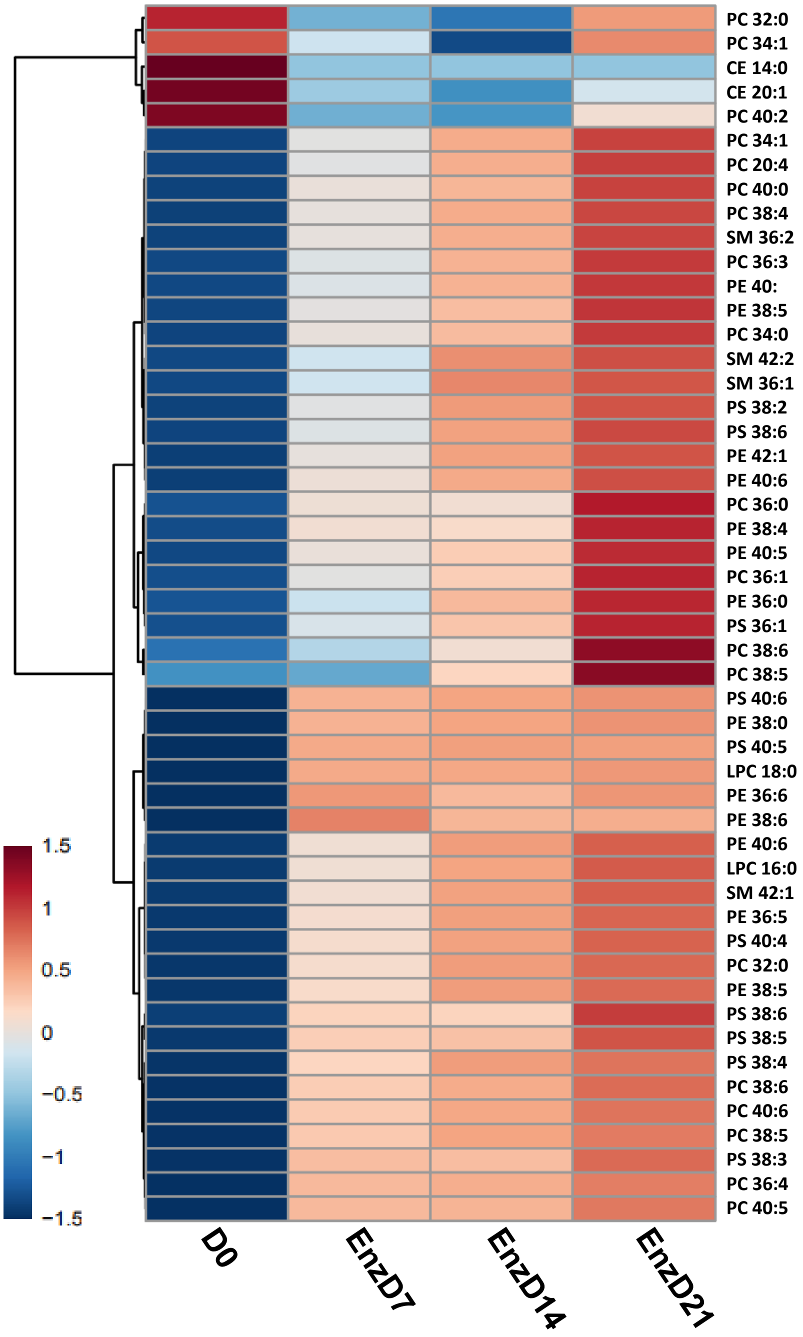

PC 32:0
PC 34:1
CE 14:0
CE 20:1
PC 40:2
PC 34:1
PC 20:4
PC 40:0
PC 38:4
SM 36:2
PC 36:3
PE 40:
PE 38:5
PC 34:0
SM 42:2
SM 36:1
PS 38:2
PS 38:6
PE 42:1
PE 40:6
PC 36:0
PE 38:4
PE 40:5
PC 36:1
PE 36:0
PS 36:1
PC 38:6
PC 38:5
PS 40:6
PE 38:0
PS 40:5
LPC 18:0
PE 36:6
PE 38:6
PE 40:6
LPC 16:0
SM 42:1
PE 36:5
PS 40:4
PC 32:0
PE 38:5
PS 38:6
PS 38:5
PS 38:4
PC 38:6
PC 40:6
PC 38:5
PS 38:3
PC 36:4
PC 40:5
1.5
1
0.5
0
−0.5
−1
−1.5
D0
EnzD7
EnzD14
EnzD21

Fig S4A

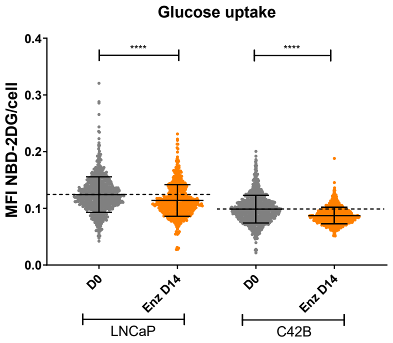

Fig S4B

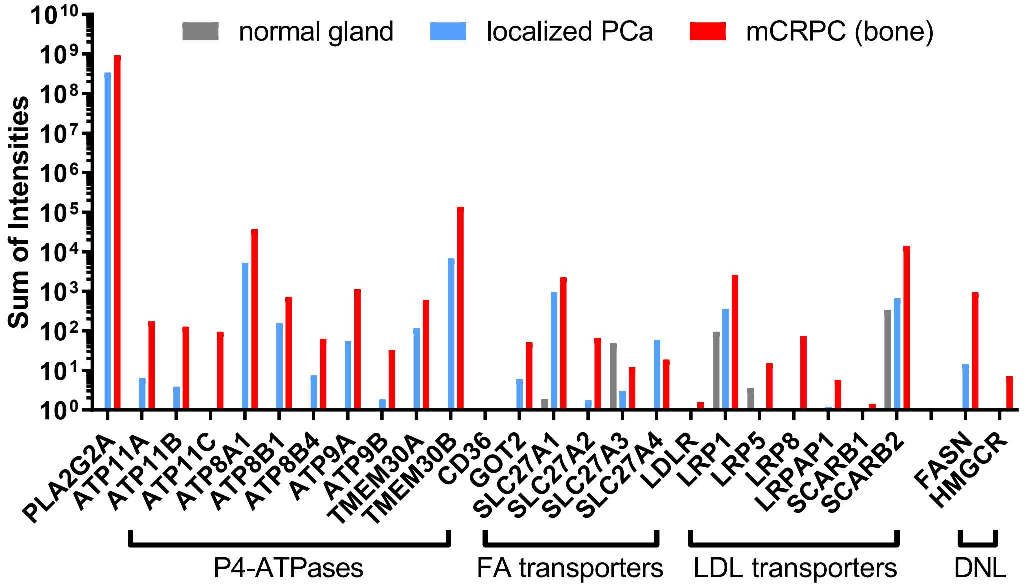

Fig S4C

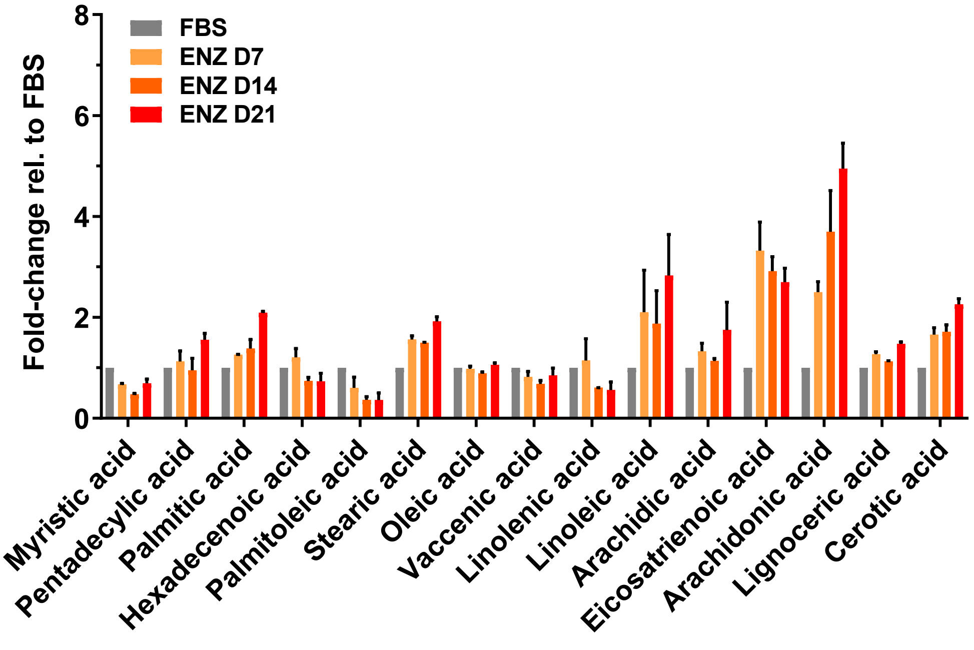

Fig S4D

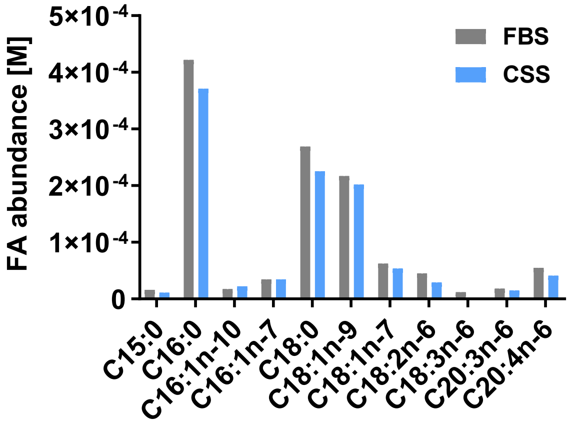

	FBS	CSS
cholesterol	34 mg/dL	36 mg/dL
TAGs	60 mg/dL	66 mg/dL

Fig S4E

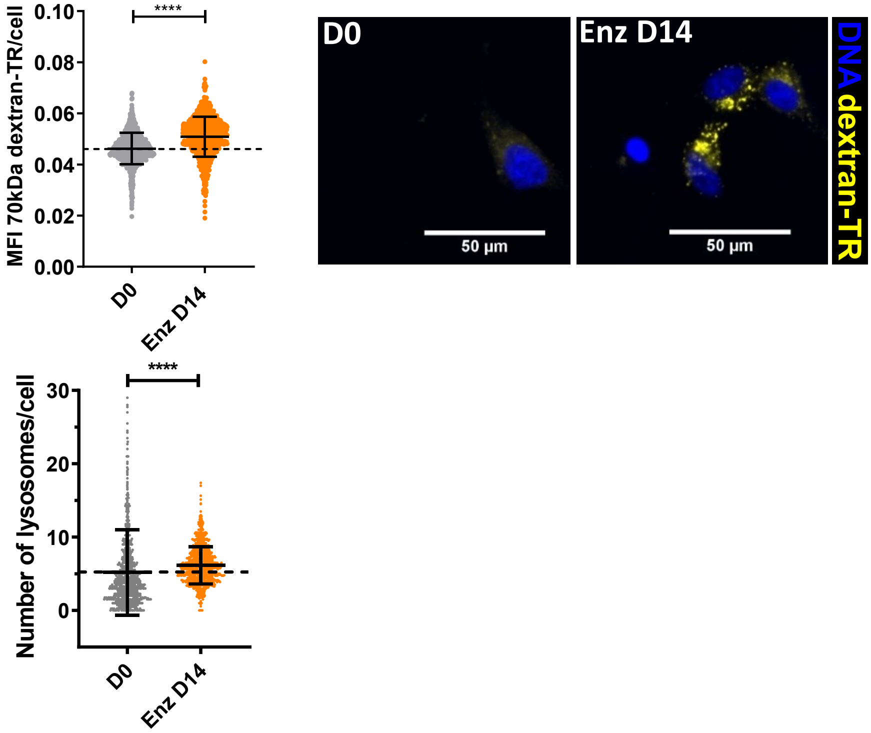

Fig S4F

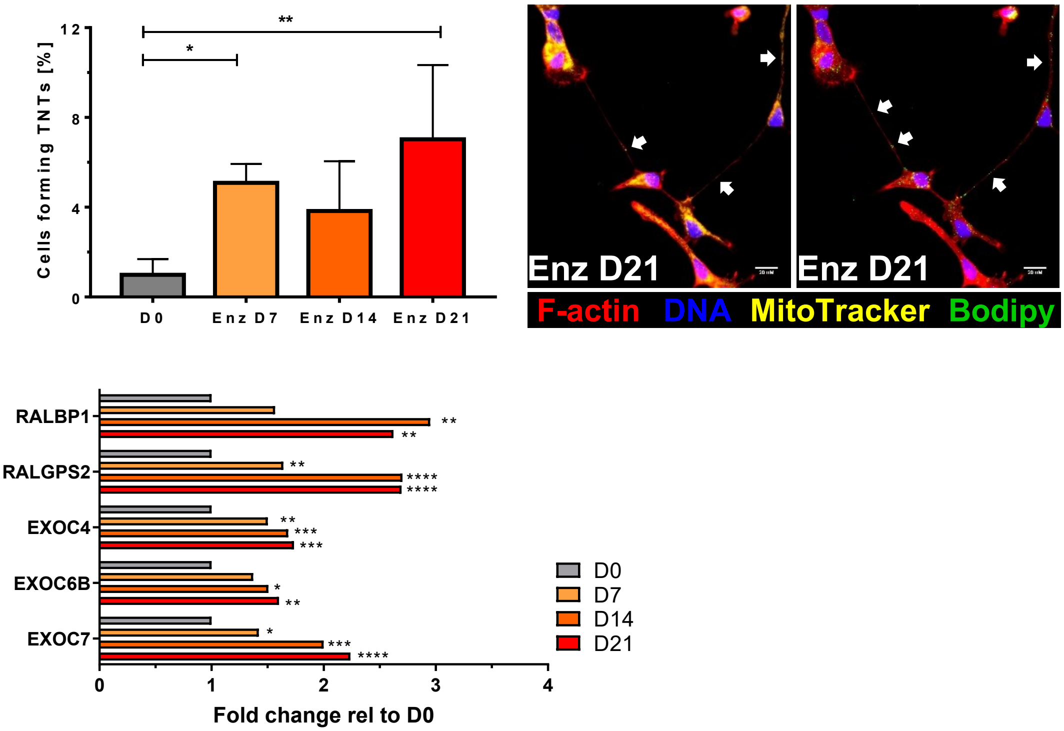

Fig S5A

KEGG pathway name	Number of genes	P-value
Arachidonic acid metabolism	13	2.34e-10
Linoleic acid metabolism	9	2.86e-10
Retinol metabolism	8	0.000011
Biosynthesis of unsaturated fatty acids	5	0.000025
Drug Metabolism	7	0.0015
Metabolism of xenobiotics	7	0.00253
Fatty acid metabolism	4	0.0267
Fatty acid elongation	3	0.0435

KEGG pathway name	Number of proteins	P-value
**Biosynthesis of unsaturated fatty acids**	**5**	**3.99e-5**
**Glycosphingolipid biosynthesis**	**4**	**8.95e-4**
**Fatty acid metabolism**	**4**	**0.0062**
Fatty acid elongation	1	0.0605

Fig S5B

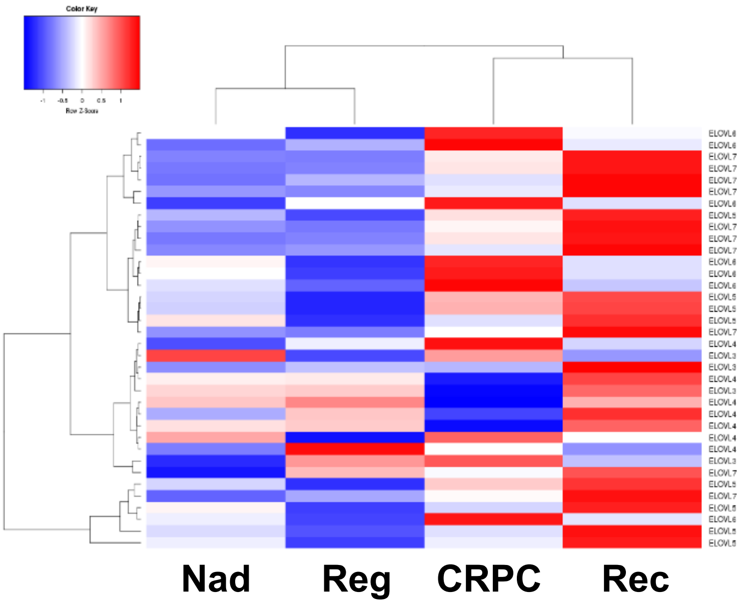

Fig S5C

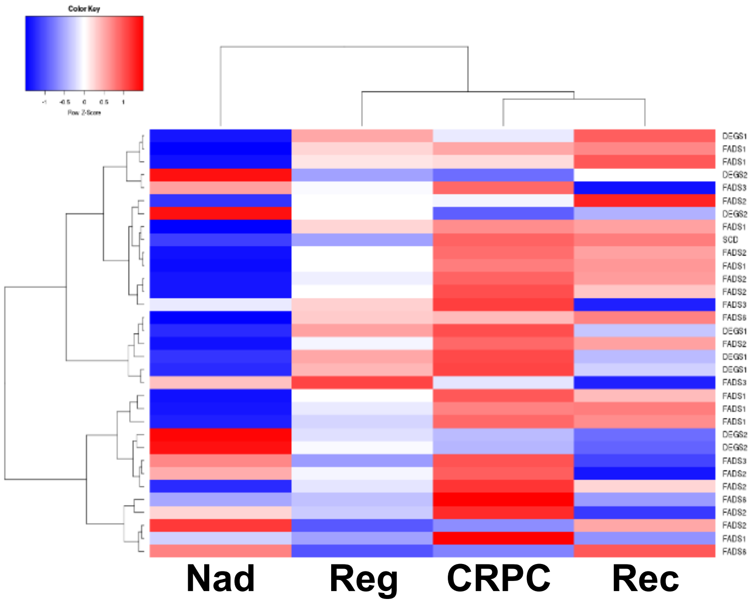

Fig S5D

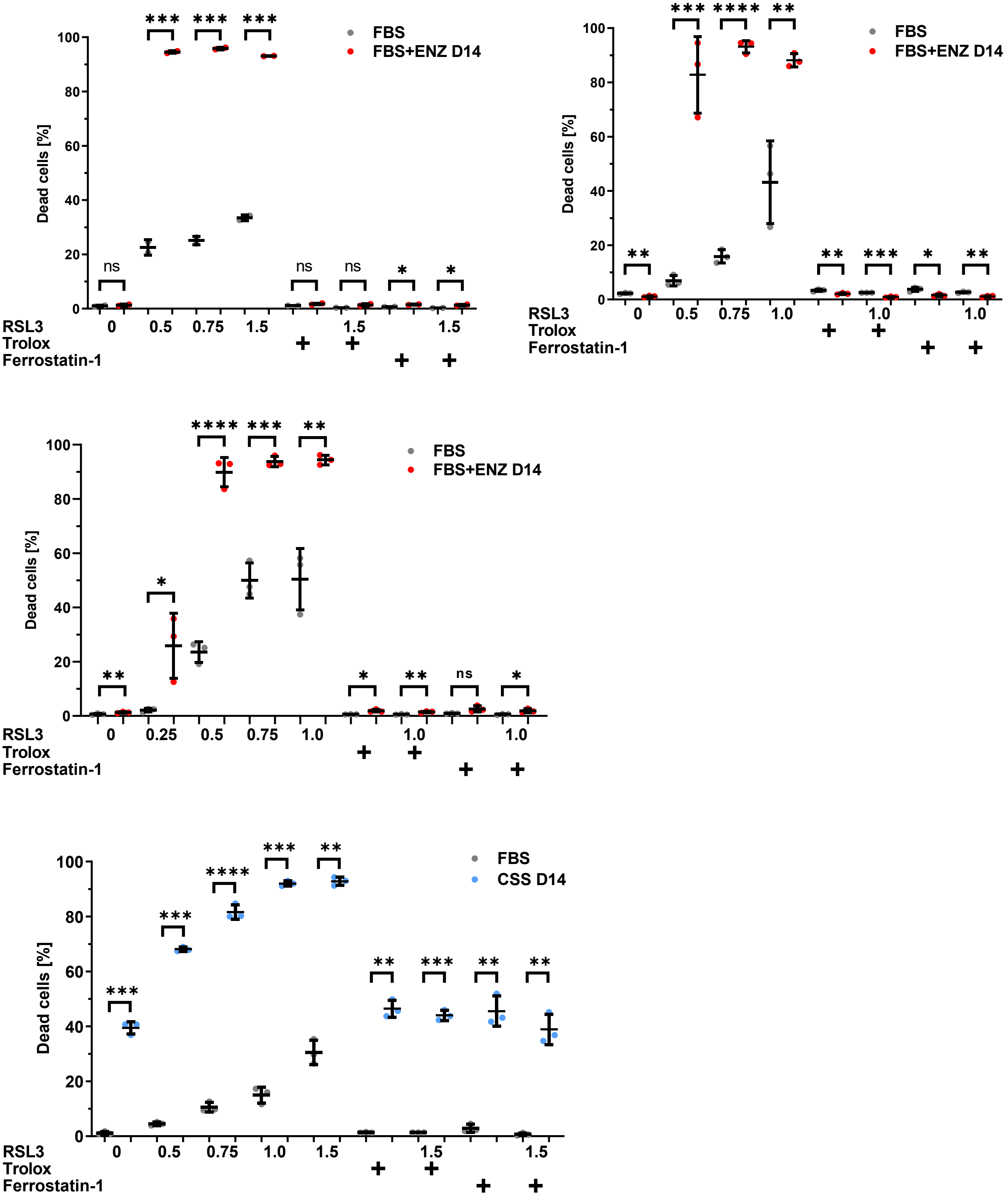
FBS
FBS+ENZ D14
Dead cells [%]
RSL3
Trolox
Ferrostatin-1
0
0.5
0.75
1.5
ns
*
***
FBS
FBS+ENZ D14
Dead cells [%]
RSL3
Trolox
Ferrostatin-1
0
0.5
0.75
1.0
**
***
****
*
FBS
FBS+ENZ D14
Dead cells [%]
RSL3
Trolox
Ferrostatin-1
0
0.25
0.5
0.75
1.0
FBS
CSS D14
Dead cells [%]
RSL3
Trolox
Ferrostatin-1
0
0.5
0.75
1.0
1.5

Fig S5E

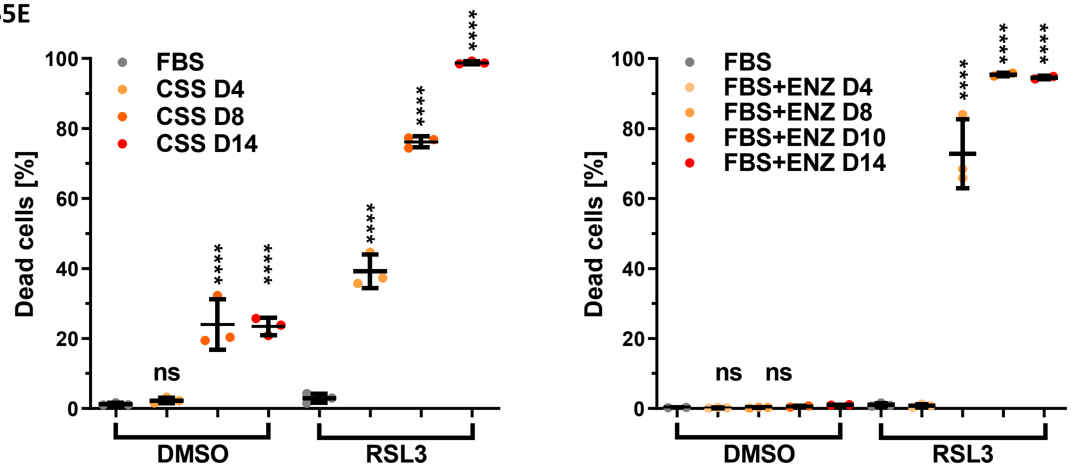

Fig S5F

Reactome pathways	# reference list	# detected	# expected	+/-	Fold Enrichment	raw P value	FDR
Selenocysteine synthesis (R-HSA-2408557)	93	51	1.48	+	34.47	1.48E-55	2.70E-53
Selenoamino acid metabolism (R-HSA-2408522	116	51	1.85	+	27.64	1.01E-51	1.30E-49
eNOS activation (R-HSA-203615)	11	3	0.17	+	17.14	1.22E-03	2.47E-02
Metabolism of nitric oxide (R-HSA-202131)	15	3	0.24	+	12.57	2.61E-03	4.23E-02
eNOS activation and regulation (R-HSA-203765	15	3	0.24	+	12.57	2.61E-03	4.17E-02
Cellular responses to stress (R-HSA-2262752)	393	19	6.25	+	3.04	3.01E-05	9.55E-04

Fig S5G

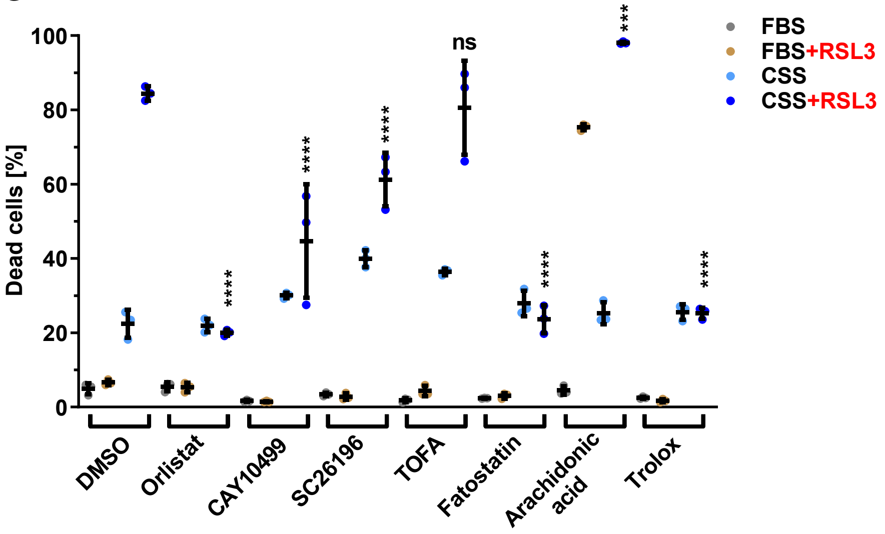

Supplement: Supplementary file 2 — Additional file 2. Supplementary Figures. [file 40170_2020_217_MOESM2_ESM.pdf]
